# Discovery of Novel Tofacitinib–ADTOH Molecular Hybridization Derivatives for the Treatment of Ulcerative Colitis

**DOI:** 10.3390/antiox14030325

**Published:** 2025-03-08

**Authors:** Yi Mou, Shuai Wen, Yan Wang, Yao Zhao, Ying-Ping Li, Hong-Kai Sha, Li-Juan Gui, Zheng-Yu Jiang, Xiang-Ming Xu

**Affiliations:** 1 College of Pharmacy and Chemistry & Chemical Engineering, Taizhou University, Taizhou 225300, China; mouyicpu@163.com (Y.M.); wen15298503462@126.com (S.W.); wangyan20221122@163.com (Y.W.); 15996256893@163.com (Y.Z.); 2024110049@tzu.edu.cn (Y.-P.L.); shahongkaitzxy@163.com (H.-K.S.); guilijuan12@163.com (L.-J.G.); 2 Jiangsu Key Laboratory of Drug Design and Optimization, China Pharmaceutical University, Nanjing 210009, China

**Keywords:** ulcerative colitis, UC therapeutic drugs, molecular hybridization, tofacitinib, ADTOH

## Abstract

The treatment of ulcerative colitis (UC) has been a major medical challenge due to the lack of safe and effective drugs. Molecular hybridization is a promising strategy for the development of drugs with pleiotropic activity, which has been demonstrated in a wide range of diseases. Tofacitinib has exhibited significant effects on the remission of UC, but a series of adverse effects have occurred during its clinical application. Herein, we propose to utilize a molecular hybridization strategy to link tofacitinib with a cytoprotective H_2_S donor (ADTOH) to obtain a series of hybridized molecules **ZX-4C**~**ZX-6C**. Among them, **ZX-4C** exhibited the best performance in the H_2_S release rate and the cytoprotective effects against dextran sulfate sodium (DSS)-induced injury. The in vivo studies showed that **ZX-4C** could effectively alleviate DSS-induced colitis by enhancing oxidative stress defense and reducing the inflammatory response, demonstrating that it is more potent than the parent drugs. The data from the present study support that this molecular hybridization strategy provides a promising avenue for the treatment of UC.

## 1. Introduction

Ulcerative colitis (UC) is a chronic non-specific inflammatory bowel disease (IBD). In recent years, there has been a rising incidence of UC, significantly impacting the quality of life for patients [1,2,3,4]. The treatment of UC has become a major medical challenge, due to the complexities associated with its treatment [5,6]. Conventional pharmacotherapies include aminosalicylic acid preparations [7,8], steroid hormones [9], and immunosuppressants [10]. These drugs have some efficacy in the treatment of UC. However, some serious side effects may limit their wide clinical application, such as gastrointestinal reactions and changes in the blood system [11,12]. The JAK/STAT pathway is often associated with a variety of inflammatory cytokine responses, and thus JAK could be potential therapeutic targets for UC [13,14,15]. In recent years, some JAK inhibitors, such as tofacitinib and filgotinib, have been successfully approved for the treatment of UC [15]. Clinical evidence indicates that tofacitinib has a significant effect on the remission of UC, especially in patients with insufficient response to conventional treatment, and it has become a commonly used drug for the treatment of UC [16,17]. However, a series of adverse effects occur during the clinical use of tofacitinib, such as reduced hemoglobin, decreased absolute neutrophil count, increased total cholesterol, and infection [18]. More seriously, the FDA has recently issued a black-box warning about an increased risk of thrombosis and death with tofacitinib (10 mg twice daily) [19,20]. Therefore, a rational drug design strategy to develop tofacitinib derivatives is essential to reduce the adverse events of tofacitinib and improve its efficacy in UC [21,22]. For example, some tofacitinib prodrugs have been successfully designed for the treatment of UC and have achieved some promising results [23].

**Figure 1:**
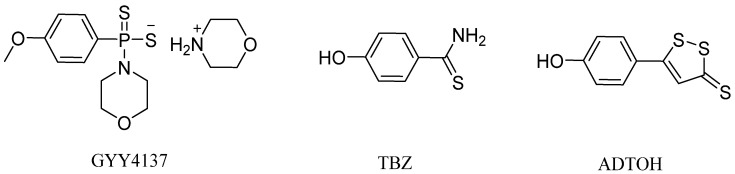
Structures of representative synthetic H_2_S donors.

As an important endogenous gas signaling molecule, hydrogen sulfide (H_2_S) has been demonstrated to be an important mediator of gastrointestinal mucosal defense, contributing to the repair of damaged tissues and promoting the resolution of colitis [24,25]. Studies have revealed that H_2_S alleviates colitis symptoms by attenuating the expression of many pro-inflammatory cytokines [26,27]. H_2_S donors are generally a class of small molecules that can release H_2_S, and serve not only as valuable research tools but also as potential therapeutic agents. Studies have confirmed that H2S donors exert anti-inflammatory effects through a variety of pathways, including inhibition of leukocyte transfer and reduction in inflammatory mediator production, and that they can reduce the production and release of cytokines, thereby reducing tissue damage and promoting inflammation to subside. Various H_2_S donors, such as GYY4137, 4-hydroxythiobenzamide (4-OH-TBZ), 5-(4-hydroxyphenyl)-3H-1,2-dithio-3-thioone (ADTOH), etc. (Figure 1), have been developed and intensely investigated [28,29,30]. Research has established that an H2S donor can be covalently incorporated into the structure of bioactive drugs to obtain new hybrid compounds, and this strategy has been proven beneficial in reducing the toxic side effects of the parent drug [31]. The covalent conjugation of H₂S donors with non-steroidal anti-inflammatory drugs (NSAIDs) has been demonstrated to remarkably reduce gastrointestinal toxicity and enhance therapeutic efficacy [32,33]. Specifically, ATB-352, which is a hybrid of ADTOH and ketoprofen, has attracted substantial research attention. Studies have shown that ATB-352 not only maintained anti-inflammatory activity similar to ketoprofen but also significantly reduced gastrointestinal toxicity [34].

Clinically, the combination of specific drugs represents an important strategy for the treatment of UC [35]. However, it is difficult to balance the safety and efficacy of the drug cocktail therapy due to the disadvantages of drug–drug interactions and poor patient compliance [36]. Molecular hybridization is an emerging concept in drug design and development which can produce novel hybrid compounds with improved affinity and efficacy based on the combination of pharmacophores of different bioactive substances in contrast to the parent drug. Molecular hybridization strategies enhance the rate and extent of drug absorption in the body by optimizing the physicochemical properties of the drug (molecular size, lipid solubility, etc.); drugs designed by molecular hybridization can better control the metabolism and absorption of drugs in vivo, reduce the first-pass effect to some extent, and improve bioavailability. Several studies have demonstrated that molecular hybridization is a promising strategy for the development of pleiotropic drugs for multifactorial diseases of unknown etiology, such as UC [37]. Considering that H_2_S donor hybrids help reduce the toxic side effects of parent drugs, we designed and prepared a series of new hybrid molecules by coupling tofacitinib with the H_2_S donor ADTOH. These hybrid molecules showed significant therapeutic effects on UC in in vivo and in vitro dextran sodium sulfate (DSS)-induced experimental colitis.

## 2. Experimental Section

### 2.1. Chemistry Section

All commercially available chemicals are used directly without purification. Reactions were monitored by thin-layer chromatography and visualized under UV light. ^1^H NMR and ^13^CNMR spectra were recorded on a Bruker Avance-300 instrument with CDCl_3_ or DMSO-*d*_6_. High-resolution mass spectra were obtained on an Agilent 6230 TOF. The LC-20AT protruding liquid chromatography system (Shimadzu, Kyoto, Japan) was used, and the column was a Shimadzu C18 (4.6 mm × 150 mm, 3.5 μm). The mobile phase was the solvent methanol/water 80:20, TFA 1‰. The flow rate was 1 mL/min, and the ultraviolet detector SPD-20A was used with a wavelength of 254 nm.

#### 2.1.1. General Synthetic Procedure A for Intermediates **3a**–**c**

ADTOH (1.00 equiv) was solubilized with DMF (10 mL), K_2_CO_3_ (2.00 equiv) was added, followed by the addition of **2a**–**c** (1.00 equiv) solution of DMF, and the reaction was carried out at room temperature for 12h. The complete reaction was monitored by TLC, and the reaction solution was added with water and extracted by EtOAc. The organic phases were combined, dried over anhydrous sodium sulfate, and concentrated. Silica gel column chromatography purification afforded intermediates **3a**–**c**.

Tert-butyl 4-(4-(3-thioxo-3H-1,2-dithiol-5-yl)phenoxy)butanoate (**3a**). Synthesized following procedure A, yellow solid, yield: 76.2%. ^1^H NMR (300 MHz, DMSO-*d*_6_) δ 7.96–7.85 (m, 2H), 7.78 (s, 1H), 7.14–7.04 (m, 2H), 4.10 (t, *J* = 5.7 Hz, 2H), 2.39 (t, *J* = 7.3 Hz, 2H), 2.06–1.90 (m, 2H), 1.42 (s, 9H).

Tert-butyl 5-(4-(3-thioxo-3H-1,2-dithiol-5-yl)phenoxy)pentanoate (**3b**). Synthesized following procedure A, yellow solid, yield: 82.1%. ^1^H NMR (300 MHz, Chloroform-*d*) δ 7.62–7.59 (m, 2H), 7.40 (s, 1H), 6.97–6.95 (m, 2H), 4.04 (t, *J* = 5.9 Hz, 2H), 2.32 (t, *J* = 6.9 Hz, 2H), 1.88–1.83 (m, 2H), 1.82–1.78 (m, 2H), 1.46 (s, 9H).

Tert-butyl 6-(4-(3-thioxo-3H-1,2-dithiol-5-yl)phenoxy)hexanoate (**3c**). Synthesized following procedure A, yellow solid, yield: 81.0%. ^1^H NMR (300 MHz, Chloroform-*d*) δ 7.66–7.52 (m, 2H), 7.37 (s, 1H), 7.01–6.89 (m, 2H), 4.02 (t, *J* = 6.4 Hz, 2H), 2.26 (t, *J* = 7.3 Hz, 2H), 1.86–1.79 (m, 2H), 1.70–1.62 (m, 2H), 1.57–1.48 (m, 2H), 1.45 (s, 9H).

#### 2.1.2. General Synthetic Procedure B for Intermediates **4a**–**c**

Trifluoroacetic acid was added to the DCM (20 mL) solution of the intermediate **3a**–**c** (1.00 equiv). The reaction solution was stirred at room temperature for 4 h. The complete reaction was monitored by TLC, which concentrated the reaction solution. The crude product was separated and purified by column chromatography to obtain intermediates **4a**–**c**.

4-(4-(3-thioxo-3H-1,2-dithiol-5-yl)phenoxy)butanoic acid (**4a**). Synthesized following procedure B, yellow solid, yield: 84.3%. ^1^H NMR (300 MHz, DMSO-*d*_6_) δ 12.16 (s, 1H), 7.92–7.83 (m, 2H), 7.76 (s, 1H), 7.13–7.02 (m, 2H), 4.09 (t, *J* = 6.4 Hz, 2H), 2.40 (t, *J* = 7.3 Hz, 2H), 1.99–1.94 (m, 2H).

5-(4-(3-thioxo-3H-1,2-dithiol-5-yl)phenoxy)pentanoic acid (**4b**). Synthesized following procedure B, yellow solid, yield: 85.4%. ^1^H NMR (300 MHz, DMSO-*d*_6_) δ 12.08 (s, 1H), 7.92–7.84 (m, 2H), 7.78 (s, 1H), 7.12–7.04 (m, 2H), 4.08 (t, *J* = 6.1 Hz, 2H), 2.30 (t, *J* = 7.1 Hz, 2H), 1.82–1.71 (m, 2H), 1.70–1.60 (m, 2H).

6-(4-(3-thioxo-3H-1,2-dithiol-5-yl)phenoxy)hexanoic acid (**4c**). Synthesized following procedure B, yellow solid, yield: 82.4%. ^1^H NMR (300 MHz, DMSO-*d*_6_) δ 12.04 (s, 1H), 7.92–7.85 (m, 2H), 7.79 (s, 1H), 7.13–7.04 (m, 2H), 4.08 (t, *J* = 6.5 Hz, 2H), 2.25 (t, *J* = 7.2 Hz, 2H), 1.80–171 (m, 2H), 1.64–1.52 (m, 2H), 1.50–1.38 (m, 2H).

#### 2.1.3. Synthetic Procedure for Intermediate **7**

Ingredient **5** (1882.0 mg, 10.0 mmol) was dissolved with DCM, followed by the addition of ingredient **6** (1410.0 mg, 10.0 mmol) and DIPEA (2585.0 mg, 20.0 mmol) in an ice bath. The reaction mixture was stirred at room temperature for 12 h under nitrogen. The complete reaction was monitored by TLC, which concentrated the reaction solution. The crude product was separated and purified by column chromatography to obtain intermediate **7** as a light yellow oil, yielding 70.2%. ^1^H NMR (300 MHz, Chloroform-*d*) δ 6.63–6.53 (m, 1H), 3.51–3.25 (m, 4H), 2.98–2.96 (m, 3H), 2.89 (s, 3H), 1.83–1.79 (m, 3H), 1.45 (s, 9H).

#### 2.1.4. General Synthetic Procedure C for Intermediates **8a**–**c**

Intermediates **4a**–**c** (1.00 equiv) were dissolved with DMF (10 mL), followed by the addition of intermediate **7** (1.00 equiv) and K_2_CO_3_ (1.10 equiv). The reaction mixture was stirred at room temperature for 2 h. The complete reaction was monitored by TLC, and the reaction solution was added with water and extracted by EtOAc. The organic phases were combined, dried over anhydrous sodium sulfate, and concentrated. Silica gel column chromatography purification afforded intermediates **8a**–**c**.

5,8,11,11-tetramethyl-4,9-dioxo-3,10-dioxa-5,8-diazadodecan-2-yl 4-(4-(3-thioxo-3H-1,2-dithiol-5-yl)phenoxy)butanoate (**8a**). Synthesized following procedure C, yellow solid, yield: 89.3%. ^1^H NMR (300 MHz, Chloroform-*d*) δ 7.64–7.55 (m, 2H), 7.38 (s, 1H), 7.02–6.92 (m, 2H), 6.89–6.78 (m, 1H), 4.08 (t, *J* = 6.1 Hz, 2H), 3.39–3.29 (m, 4H), 2.93 (s, 3H), 2.86 (s, 3H), 2.57–2.52 (m, 2H), 2.16–2.12 (m, 2H), 1.53–1.47 (m, 3H), 1.45 (s, 9H).

5,8,11,11-tetramethyl-4,9-dioxo-3,10-dioxa-5,8-diazadodecan-2-yl 5-(4-(3-thioxo-3H-1,2-dithiol-5-yl)phenoxy)pentanoate (**8b**). Synthesized following procedure C, yellow solid, yield: 83.3%. ^1^H NMR (300 MHz, Chloroform-*d*) δ 7.65–7.55 (m, 2H), 7.39 (s, 1H), 6.97–6.94 (m, 2H), 6.85-6.80 (m, 1H), 4.04 (t, *J* = 5.4 Hz, 2H), 3.44-3.30 (m, 4H), 2.94 (s, 3H), 2.87 (s, 3H), 2.53–2.33 (m, 2H), 1.89–1.85 (m, 2H), 1.84–1.78 (m, 2H), 1.53–1.49 (m, 3H), 1.46 (s, 9H).

5,8,11,11-tetramethyl-4,9-dioxo-3,10-dioxa-5,8-diazadodecan-2-yl 6-(4-(3-thioxo-3H-1,2-dithiol-5-yl)phenoxy)hexanoate (**8c**). Synthesized following procedure C, yellow solid, yield: 80.3%. ^1^H NMR (300 MHz, Chloroform-*d*) δ 7.62–7.58 (m, 2H), 7.38 (s, 1H), 7.00–6.91 (m, 2H), 6.84–6.80 (m, 1H), 4.02 (t, *J* = 6.4 Hz, 2H), 3.50–3.24 (m, 4H), 2.94 (s, 3H), 2.88 (s, 3H), 2.39–2.34 (m, 2H), 1.88–1.79 (m, 2H), 1.77–1.67 (m, 2H), 1.57–1.52 (m, 2H), 1.51–1.48 (m, 3H), 1.46 (s, 9H).

#### 2.1.5. General Synthetic Procedure D for Intermediates **9a**–**c**

Intermediates **8a**–**c** (1.00 equiv) were dissolved with EtOAc (10 mL), followed by the addition of an HCl solution. The reaction mixture was stirred at room temperature for 4 h. The complete reaction was monitored by TLC, which concentrated the reaction solution. The crude product is used directly in the next step without purification.

#### 2.1.6. Synthetic Procedure for Intermediate **12**

Ingredient **10** (312.2 mg, 1.0 mmol) was dissolved with DCM (15 mL), followed by the addition of ingredient **11** (201.0 mg, 1.0 mmol) and DIPEA (258.0 mg, 2.0 mmol) in an ice bath. The reaction mixture was stirred at room temperature for 6 h under nitrogen. The complete reaction was monitored by TLC, which concentrated the reaction solution. The crude product is used directly in the next step without purification.

#### 2.1.7. General Synthetic Procedure E for Target Compounds **ZX-4C**~**ZX-6C**

Intermediates **9a**–**c** (1.00 equiv) were dissolved with DCM (12 mL), followed by the addition of intermediates **12** (1.00 equiv) and DIPEA (2.00 equiv) in an ice bath. The reaction mixture was stirred at room temperature for 4 h under nitrogen. The complete reaction was monitored by TLC, which concentrated the reaction solution. The crude product was separated and purified by column chromatography to obtain target compounds **ZX-4C**~**ZX-6C**.

1-(((2-(4-(((3R,4R)-1-(2-cyanoacetyl)-4-methylpiperidin-3-yl) (methyl)amino)-N-methyl-7H-pyrrolo[2,3-d]pyrimidine-7-carboxamido)ethyl)(methyl)carbamoyl)oxy)ethyl 4-(4-(3-thioxo-3H-1,2-dithiol-5-yl)phenoxy)butanoate (**ZX-4C**). Synthesized following procedure E, yellow solid, yield: 48.2%. ^1^H NMR (300 MHz, Chloroform-*d*) δ 8.33–8.31 (m, 1H), 7.59 (d, *J* = 8.3 Hz, 2H), 7.39–7.37 (m, 1H), 7.20 (s, 1H), 6.95 (d, *J* = 8.3 Hz, 2H), 6.84 (s, 1H), 6.63 (s, 1H), 5.11 (s, 1H), 4.09–4.05 (m, 2H), 4.05–3.81 (m, 2H), 3.80–3.67 (m, 2H), 3.66–3.61 (m, 2H), 3.59–3.52 (m, 4H), 3.43–3.33 (m, 3H), 3.25–3.06 (m, 3H),3.05–2.70 (m, 3H), 2.55 (s, 2H), 2.52–2.43 (m, 1H), 2.18–2.12 (m, 2H), 1.76 (s, 2H), 1.55–1.44 (m, 3H), 1.12–1.07 (m, 3H); ^13^C NMR (151 MHz, CDCl_3_) δ 215.20, 173.13, 171.19, 162.28, 160.14, 157.77, 151.90, 134.68, 128.65, 124.26, 115.56, 113.80, 104.43, 103.55, 90.10, 90.08, 67.09, 53.77, 52.93, 47.45, 46.43, 44.02, 43.03, 39.93, 35.47, 34.70, 31.70, 31.33, 30.52, 29.76, 27.28, 25.17, 24.23, 19.95, 14.66, 14.25, 11.29; HRMS (ESI): found 809.2537 (C_37_H_44_N_8_O_7_S_3_, [M + H]^+^, requires 809.2568); HPLC: t_R_ = 3.92 min, 98.126%.

1-(((2-(4-(((3R,4R)-1-(2-cyanoacetyl)-4-methylpiperidin-3-yl)(methyl)amino)-N-methyl-7H-pyrrolo[2,3-d]pyrimidine-7-carboxamido)ethyl)(methyl)carbamoyl)oxy)ethyl 5-(4-(3-thioxo-3H-1,2-dithiol-5-yl)phenoxy)pentanoate (**ZX-5C**). Synthesized following procedure E, yellow solid, yield: 41.6%. ^1^H NMR (300 MHz, Chloroform-*d*) δ 8.33–8.32 (m, 1H), 7.63–7.55 (m, 2H), 7.39–7.38 (m, 1H), 7.24–7.13 (m, 1H), 6.94 (d, *J* = 8.4 Hz, 2H), 6.84–6.82 (m, 1H), 6.63–6.62 (m, 1H), 5.11 (s, 1H), 4.09–4.04 (m, 2H), 4.03–3.81 (m, 2H), 3.79–3.67 (m, 2H), 3.64–3.61 (m, 2H), 3.59–3.50 (m, 4H), 3.40–3.35 (m, 3H), 3.25–3.05 (m, 3H), 3.03–2.55 (m, 3H), 2.53–2.45 (m, 2H), 2.42–2.32 (m, 1H), 2.10–1.90 (m, 2H) 1.88–1.85 (m, 2H), 1.84–1.80 (m, 2H), 1.55–1.45 (m, 3H), 1.12–1.07 (m, 3H); ^13^C NMR (75 MHz, CDCl_3_) δ 215.15, 173.30, 171.51, 162.44, 160.15, 157.77, 151.96, 134.63, 128.67, 124.10, 122.51, 115.51, 113.87, 104.45, 103.55, 90.01, 67.90, 53.72, 52.91, 46.45, 43.98, 42.99, 39.90, 35.44, 34.70, 33.71, 31.66, 31.33, 30.47, 30.26, 29.78, 29.41, 28.38, 25.21, 21.31, 19.97, 14.64, 14.23; HRMS (ESI): found 845.2513 (C_38_H_46_N_8_O_7_S_3_, [M + Na]^+^, requires 845.2549); HPLC: t_R_ = 4.36 min, 98.946%.

1-(((2-(4-(((3R,4R)-1-(2-cyanoacetyl)-4-methylpiperidin-3-yl)(methyl)amino)-N-methyl-7H-pyrrolo[2,3-d]pyrimidine-7-carboxamido)ethyl)(methyl)carbamoyl)oxy)ethyl 6-(4-(3-thioxo-3H-1,2-dithiol-5-yl)phenoxy)hexanoate (**ZX-6C**). Synthesized following procedure E, yellow solid, yield: 39.6%. ^1^H NMR (300 MHz, Chloroform-*d*) δ 8.33–8.31 (m, 1H), 7.65–7.55 (m, 2H), 7.44–7.36 (m, 1H), 7.22–7.16 (m, 1H), 6.94 (d, *J* = 8.5 Hz, 2H), 6.83–6.76 (m, 1H), 6.65–6.62 (m, 1H), 5.10 (s, 1H), 4.10–4.01 (m, 2H), 4.00–3.75 (m, 2H), 3.74–3.65 (m, 2H), 3.63–3.57 (m, 2H), 3.56–3.45 (m, 4H), 3.41–3.33 (m, 3H), 3.23–3.05 (m, 3H), 3.04–2.55 (m, 3H), 2.53–2.37 (m, 2H), 2.36–2.33 (m, 1H), 2.05–1.85 (m, 2H), 1.83–1.78 (m, 2H), 1.77–1.71 (m, 2H), 1.70–1.60 (m, 2H), 1.55–1.45 (m, 3H), 1.12–1.07 (m, 3H); ^13^C NMR (75 MHz, CDCl_3_) δ 215.07, 173.27, 171.63, 162.50, 160.13, 157.62, 151.77, 134.54, 128.62, 123.97, 122.54, 115.46, 113.86, 104.44, 103.48, 89.91, 68.11, 53.75, 52.96, 47.26, 46.38, 43.89, 42.89, 39.80, 35.40, 34.69, 33.99, 31.61, 31.27, 30.42, 29.73, 28.72, 25.44, 25.17, 24.31, 22.71, 19.92, 14.56, 14.17; HRMS (ESI): found 837.2852 (C_39_H_48_N_8_O_7_S_3_, [M + H]^+^, requires 837.2881); HPLC: t_R_ = 5.09 min, 96.471%.

### 2.2. Evaluation of Hydrogen Sulfide Release

Determination of H_2_S release from compounds using the methylene blue method. The target compounds were dissolved in DMSO as 10 mM solutions, then diluted into an incubation solution with a final concentration of 200 μM using phosphate-buffered solution (pH 7.4, containing 1 mM TCEP). Seal the solution and incubate at 37 °C. At regular intervals, 200 μL of reaction solution was pipetted into a 1.5 mL epitube using a commercially available kit (H_2_S Content Determination Kit, YX-C-C000, Sinobestbio, Shanghai, China) according to the instructions. The experiment was repeated three times in parallel. The H_2_S concentration was calculated from the standard curve of Na_2_S, and the curve of H_2_S concentration with time was plotted.

### 2.3. Fluorescent Probe to Study H_2_S Release in Cultured Cells

To study the release of H_2_S from cultured cells using the fluorescent probe WSP-5, NCM460 cells were inoculated in 12-well plates and incubated overnight. The compounds were prepared in culture medium to a final concentration of 50 μM, and different groups were set up, and then added to NCM460 cells. The cells were then incubated with the compounds at 37 °C for 45 min. Removed the culture medium and washed the cells with PBS three times. Then, the cells were incubated with WSP-5 (50 μM) in PBS at 37 °C for 10 min. Removed the PBS and washed the cells three times. Analysis was performed using a fluorescence microscope (OLYMPUS DP72, Tokyo, Japan) equipped with a U-RFL-T power supply.

### 2.4. Esterase-Responsive Drug Release as Monitored by HPLC

**ZX-4C** (final Conc. 100 μM) was added to PBS with 20 unit/mL esterase, followed by vortex mixing and incubated at 37 °C. A 200 μL reaction mixture was taken at different time points into 1.5 mL Eppendorf tubes. Then, 200 μL methanol was added, vortexed to mix, and then centrifuged at 12,000 rpm for 5 min to remove protein. The supernatant was extracted and analyzed by HPLC.

### 2.5. Cell Culture

Human NCM460 colonocytes (Nanjing KeyGen Biotech, Co., Ltd., Nanjing, China) were cultured in Roswell Park Memorial Institute (RPMI) 1640 (GiBco, Invitrogen Corp., Waltham, MA, USA) with 10% fetal bovine serum (FBS) (Gibco, Invitrogen Corp., USA) and penicillin/streptomycin at 37 °C, 5% CO_2_.

### 2.6. Cell Viability Assay

NCM460 cells were inoculated overnight in 96-well plates at a density of 1 × 10^4^ cells per well. Then, they were incubated and treated with the appropriate compounds for the specified time period. The supernatant was discarded and 100 μL of 10% CCK8 (C0038, Beyotime, Shanghai, China) solution was added to each well, and the plates were incubated at 37 °C for another 1 h. Absorbance values were measured at 450 nm using a Multiskan Spectrum Microplate Reader (Thermo Scientific, Waltham, MA, USA). For each independent experiment, the assays were performed in three replicates.

### 2.7. Detection of ROS Levels

NCM460 was seeded in 12-well plates and incubated overnight, then the cells were treated with the appropriate compounds for the indicated time. After that, the cells were washed once with PBS and stained with 10 μM DCFH-DA (S0033, Beyotime, China) in RPMI-1640 medium for 20 min at 37 °C. Analysis was conducted using a fluorescence microscope (OLYMPUS DP72, Japan) equipped with a U-RFL-T power supply.

### 2.8. Detection of GPx, SOD Activities, and GSH/GSSG Ratio

GPx (S0058, Beyotime, China) and SOD (S0101, Beyotime, China) activities and GSH/GSSG (S0053, Beyotime, China) ratio were assayed using commercially available kits according to the manufacturer’s instructions.

### 2.9. Detection of IL-1β, IL-6, and TNF-α Production

Levels of IL-1β (EK0392/EK0394, Boster, Wuhan, China), IL-6 (EK0410/EK0411, Boster, China), and TNF-α (EK0525/EK0527, Boster, China) were detected using commercially available kits according to the manufacturer’s instructions.

### 2.10. In Vivo Studies

Animal studies were carried out with reference to the protocol approved by the Institutional Animal Care and Use Committee of China Pharmaceutical University. All animals were used appropriately in a scientifically valid and ethical manner. Male C57BL/6 mice (6–8 weeks old, 17–20 g) (Gempharmatech, Nanjing, China) were acclimatized 3 days prior to experimentation.

DSS was dissolved in drinking water to a concentration of 3% (*w*/*v*). C57BL/6 mice were separated into seven groups (n = 6 per group): (1) control group, (2) DSS model group, (3) DSS + **ZX-4C** (10 mg/kg) group, (4) DSS + tofacitinib + ADTOH (10 mg/kg + 10 mg/kg) group, (5) DSS + tofacitinib (10 mg/kg) group, (6) DSS + ADTOH (10 mg/kg) group, and (7) DSS + **ZX-4C** (5 mg/kg) group. Mice in the control group were fed with normal drinking water every day, and the DSS model group was constructed by feeding with 3% (*w*/*v*) DSS solution for 7 consecutive days. The treatment groups were injected intraperitoneally with a therapeutic agent once daily for 10 days, including pre-administration 3 days prior to induction of DSS and administration for the following 7 days in conjunction with DSS exposure. During this period, mice were examined daily to record body weight, food/fluid consumption, and diarrhea and bleeding scores. At the end of the experiment, the mice were executed and the colons were collected, measured for their length, and further analyzed.

### 2.11. Histopathological Assessment

The resected colonic tissues were fixed in 10% neutral buffered formalin for 24 h. Specimens were further dehydrated and embedded in paraffin wax. To carry out a histological examination, 4 μm sections of fixed embedded colon tissues were cut and stained with H&E. Pathologists who were unaware of the treatment performed histologic assessments based on a scoring system.

### 2.12. Statistical Analysis

Data are expressed as the mean ± SEM. Statistical comparisons were performed using one-way ANOVA with Tukey’s multiple comparisons test in GraphPad Prism 8.0. Statistical significance was considered at *p* < 0.05.

## 3. Results and Discussion

### 3.1. Molecular Hybridization Strategy to Design Tofacitinib–ADTOH Hybrid Derivatives

**Figure 2:**
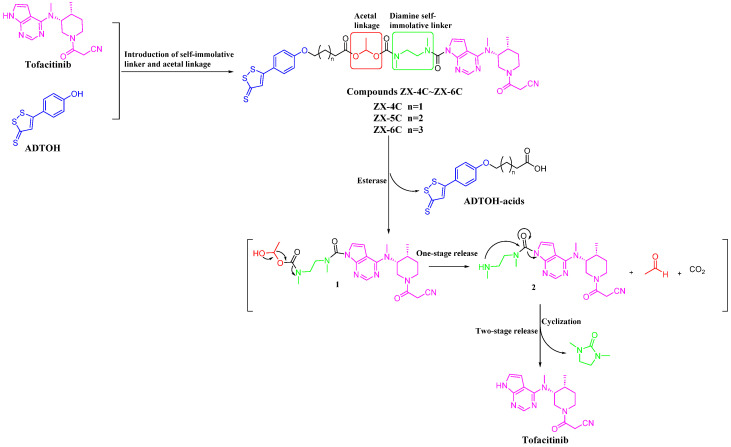
Structures and release mechanism of compounds **ZX-4C**~**ZX-6C**.

To verify the feasibility of the hybrid strategy of tofacitinib and H_2_S donor, a series of tofacitinib–ADTOH hybrid molecules were designed and synthesized. A survey of the literature revealed that the diamine moieties, as efficient self-immolation systems, had been successfully employed in several drug delivery strategies to link the PABA self-immolative moiety with payload drugs [38,39]. It has been proven that introducing diamine self-immolative linkers between the azo fragment and tofacitinib to design colon-targeted azo prodrugs was a highly effective strategy for colonic drug delivery [23]. The acetal linkages are widely used in prodrug and dual-target drug design due to their unique cleavage mechanism [40], and the acetal bond-based prodrug design is a very convenient tool for the systemic delivery of parent drugs [41]. The introduction of acetal linkage allows for multistage drug release, resulting in improved physicochemical properties and increased drug deposition and colonic absorption [42]. Finally, we introduced the acetal linkage and diamine self-immolative linkers to assemble tofacitinib and ADTOH to give a series of tofacitinib–ADTOH hybrid molecules. The proposed disassembly mechanisms of these hybrid molecules according to the related literature are shown in Figure 2 [23]. The esterase-mediated cleavage of the ester bond in compounds **ZX-4C**~**ZX-6C** releases ADTOH-acid intermediates and intermediate **1**. Then, intermediate **1** spontaneously releases to generate intermediate **2**, carbon dioxide, and acetaldehyde. Intermediate **2** contains diamine moieties, an efficient self-immolation system, then further releases to generate the corresponding 1,3-dimethyl-2-imidazolidinone and tofacitinib (Figure 2). The hybrid molecule **ZX-4C** first releases the active metabolite tofacitinib via esterase-mediated activation, followed by its subsequent entry into the conventional metabolic pathways of tofacitinib. Compared to the direct biotransformation pathway of tofacitinib, this hybrid strategy introduces a critical enzyme-controlled activation stage. Such structural modification may modulate pharmacokinetic parameters, including drug elimination half-life, plasma concentration, and bioavailability. Furthermore, by altering the rate and extent of drug metabolism to optimize pharmacokinetic properties, this approach could thereby influence therapeutic efficacy and safety profiles through refined control of systemic exposure dynamics.

### 3.2. Chemistry

**Scheme 1:**
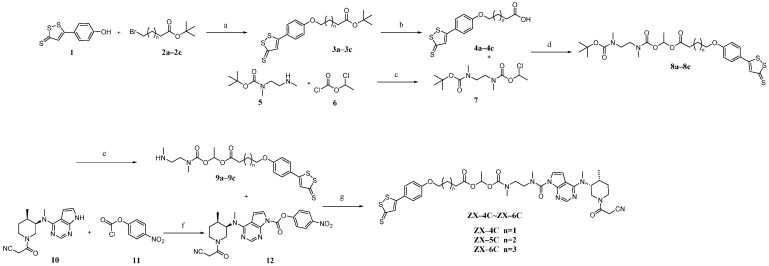
Synthesis route of ZX-4C~ZX-6C. Reagents and conditions: (a) K_2_CO_3_, DMF, rt, 12 h; (b) TFA, DCM, rt, 4 h, (c) DIPEA, DCM, rt, 12 h; (d) K_2_CO_3_, DMF, rt, 2 h; (e) HCl solution, EA, rt, 4 h; (f) DIPEA, DCM, rt, 6 h; and (g) DIPEA, DCM, rt, 4 h.

Scheme 1 illustrates the synthesis of the target compounds **ZX-4C**~**ZX-6C**. Intermediates **3a**–**3c** were synthesized using commercially available raw materials **1** and **2a**–**2c** as starting materials as reported in the literature, followed by the removal of the tert-butyl group to form intermediates **4a**–**4c**. Intermediates **8a**–**8c** were generated through a nucleophilic substitution reaction between intermediates **4a**–**4c** and **7**. Thereafter, the Boc-protecting group was removed to afford intermediates **9a**–**9c**. Intermediates **9a**–**9c** and **12** were used for the final synthesis of the target compounds **ZX-4C**~**ZX-6C**, according to the method reported in the literature (Scheme 1).

### 3.3. H_2_S Release Efficiency of Compounds ***ZX-4C***~***ZX-6C***

**Figure 3:**
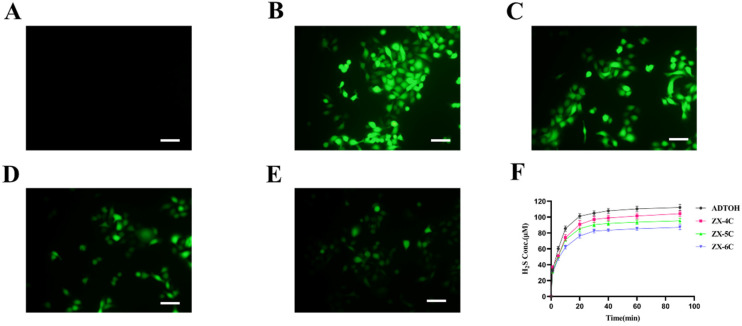
H_2_S-releasing spectrum of ADTOH and **ZX-4C**~**ZX-6C** in PBS buffer and NCM460 cells. (**A**–**E**) Detection of H_2_S release from NCM460 cells using WSP-5 fluorescent probe. Groups are divided as follows: (**A**) DMSO; (**B**) ADTOH; (**C**) **ZX-4C**; (**D**) **ZX-5C**; (**E**) **ZX-6C**. Observation of intracellular H_2_S fluorescence production under a fluorescence microscope. Scale bars, 100 μm. (**F**) H_2_S-releasing curve of ADTOH (200 μM) and **ZX-4C**~**ZX-6C** (200 μM) at 37 °C using TCEP as an accelerator. Values in (**F**) are shown as means ± SD (three independent tests).

To verify the ability of such hybrid molecules to generate hydrogen sulfide, a methylene blue assay was used. The release curves of H_2_S concentration with time are shown in Figure 3F, indicating that the generation of H_2_S increased with time, reaching a peak at about 40 min, and all tested compounds could release H_2_S in a relatively slow manner. Due to the different chemical structures, the final amount of H_2_S released from each compound is not consistent. Moreover, the length of the linker exerts a significant influence on the release of H_2_S. Specifically, an increase in the length of the linker chain corresponds to a decrease in the concentration of released H_2_S. Possibly, the large steric hindrance of the linker prevents the release of H_2_S. The larger the fragment structure, the greater the inhibition of H^2^S release. The compound **ZX-4C** demonstrated the most favorable effect, producing a relatively consistent amount of H_2_S as ADTOH, indicating that the incorporation of the tofacitinib fragment did not affect the release of H_2_S.

Having demonstrated that these hybrid molecules were able to release H_2_S in PBS buffer, we next proceeded to explore whether these compounds were able to release H_2_S in living cells. WSP-5 is a fluorescent probe for the rapid detection of H_2_S in biological samples and living cells. In this study, we utilized WSP-5 to monitor the H_2_S release of these compounds in NCM460 cells. As shown in Figure 3A–E, the NCM460 cells exhibited negligible H_2_S-derived fluorescence when no compound was added. However, with the addition of the compound, a significant fluorescence signal was produced, indicating that these hybrids could successfully produce H_2_S in the cells. The fluorescence intensity was proportional to the amount of H_2_S released. More importantly, the fluorescence intensity of all these derivatives was reduced compared with ADTOH. It was observed that an increase in the length of the linkage chain corresponded to a decrease in the concentration of H_2_S released, aligning with the H_2_S release characteristics in PBS buffer. Consequently, ZX-4C was selected for further investigation due to its superior ability to maintain H_2_S release characteristics in comparison to ADTOH.

We then evaluated the release profile of tofacitinib from **ZX-4C** in the presence of esterase. HPLC studies revealed that incubation of **ZX-4C** with esterase at 37 °C was able to generate tofacitinib within 6 h (conversion rate 85%, Supplementary Materials, Figure S1).

### 3.4. ***ZX-4C*** Exhibited Protective Effects Against DSS-Induced Injury in NCM460 Cells

**Figure 4:**
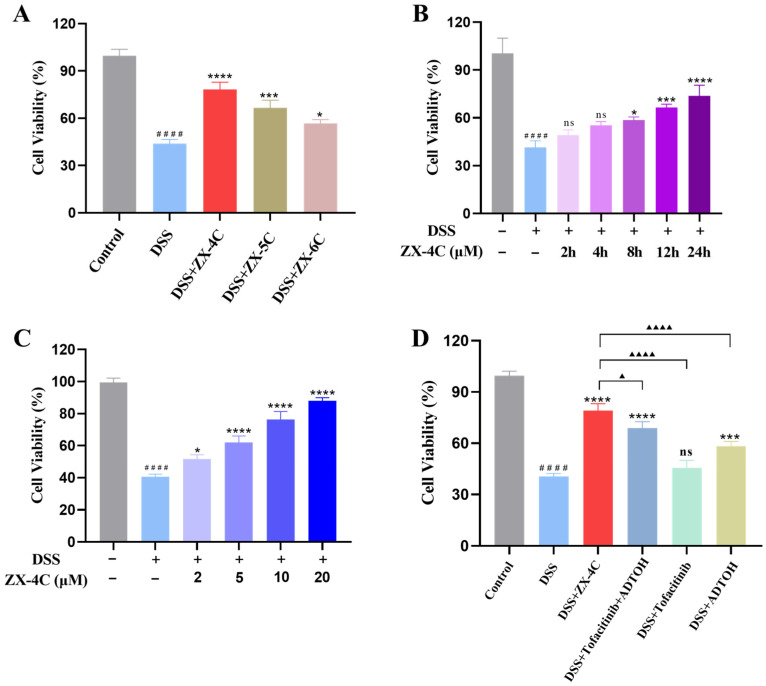
**ZX-4C** protects NCM460 cells from DSS-induced injury. (**A**) Protective effects of **ZX-4C**~**ZX-6C** against DSS-induced cell injury. NCM460 cells were pretreated with various hybrids (10 μM) for 24 h and afterward exposed to DSS (20 mg/mL) for an additional 12 h. (**B**) Time-dependent protection efficacy of **ZX-4C**. NCM460 cells were pretreatment with **ZX-4C** (10 μM) for various schedules and afterward exposed to DSS (20 mg/mL) for an additional 12 h. (**C**) Concentration-dependent protection efficacy of **ZX-4C**. NCM460 cells were pretreated with a concentration gradient of **ZX-4C** for 24 h and afterward exposed to DSS (20 mg/mL) for an additional 12 h. (**D**) **ZX-4C** displayed significant cytoprotective effects than ADTOH, tofacitinib, and tofacitinib plus ADTOH. NCM460 cells were pretreatment with 10 μM **ZX-4C**, ADTOH, tofacitinib, and tofacitinib plus ADTOH for 24 h and afterward exposed to DSS (20 mg/mL) for an additional 12 h. Values in (**A**–**D**) are shown as means ± SEM (n = 3). Statistical significance was determined by one-way ANOVA with Tukey’s multiple comparisons test: ^####^ *p* < 0.0001, vs. control group; **** *p* < 0.0001, *** *p* < 0.001, * *p* < 0.05, vs. DSS group; ^▲▲▲▲^ *p* < 0.0001, ^▲^ *p* < 0.05, vs. DSS+ **ZX-4C** group, and ns, not significant. (**A**), DSS group: *p* < 0.0001; **ZX-4C** group: *p* < 0.0001; **ZX-5C** group: *p* = 0.0002; **ZX-6C** group: *p* = 0.0149. (**B**), DSS group: *p* < 0.0001; 8 h group: *p* = 0.0128; 12 h group: *p* = 0.0004; 24 h group: *p* < 0.0001. (**C**), DSS group: *p* < 0.0001; 2 μM group: *p* = 0.011; 5 μM group: *p* < 0.0001; 10 μM group: *p* < 0.0001; 20 μM group: *p* < 0.0001. (**D**), DSS group: *p* < 0.0001; **ZX-4C** group: *p* < 0.0001; tofacitinib plus ADTOH group: *p* < 0.0001; ADTOH group: *p* = 0.0003.

UC is a chronic non-specific inflammatory bowel disease and numerous studies have demonstrated that oxidative stress plays a crucial role in the pathogenesis of UC [43]. We further investigated whether **ZX-4C** exerted therapeutic effects on UC. DSS was used to induce cellular injury of NCM460 cells. Cell Counting Kit-8 (CCK-8) assay was applied to evaluate the protective effects of hybrid molecules against DSS-induced cellular damage. As shown in Figure 4, exposure to DSS (20 mg/mL) decreased cell viability to approximately 50%. Subsequently, NCM460 cells were pretreated with **ZX-4C**~**ZX-6C** (10 μM) for 24 h, and then DSS (20 mg/mL) was added for another 12 h. All these hybrids exhibited cytoprotective effects counteracting DSS-induced injury, and **ZX-4C** demonstrated optimal protection. The corresponding cell viability was highest in this series of compounds (Figure 4A). We further validated that **ZX-4C** exhibited both time-dependent (Figure 4B) and concentration-dependent (Figure 4C) protection against DSS-induced cellular damage. Moreover, at the same concentration, **ZX-4C**, ADTOH, tofacitinib, and tofacitinib plus ADTOH all exhibited cytoprotective effects, and ADTOH showed stronger cytoprotective effects than tofacitinib. Notably, **ZX-4C** demonstrated more substantial cytoprotective effects, suggesting that the molecular hybridization combination of tofacitinib and ADTOH produced more pronounced protective effects than the drug combination (Figure 4D).

**Figure 5:**
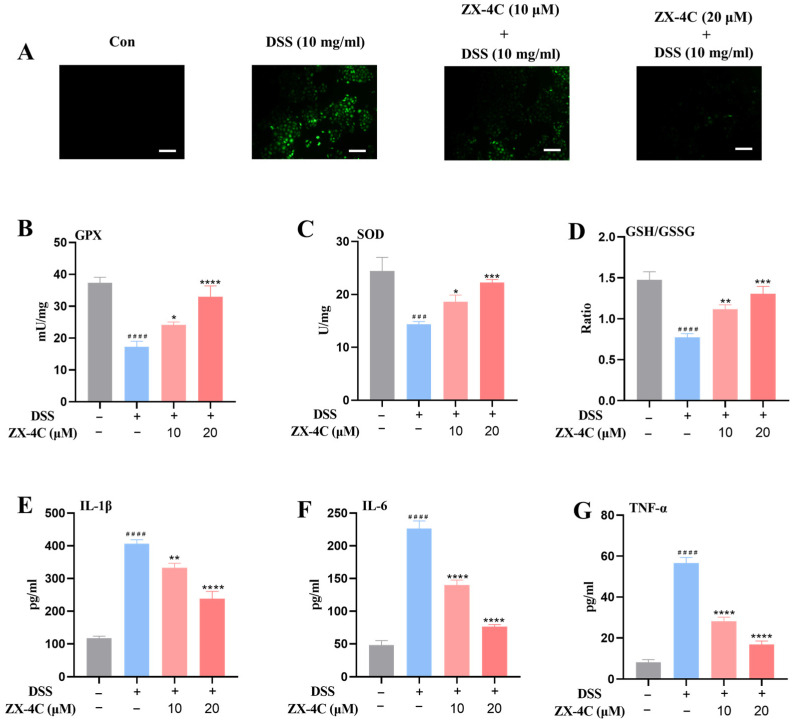
Measurement of cellular ROS levels, antioxidant enzyme activities, and pro-inflammatory cytokine levels. (**A**) Detection of cellular ROS levels in NCM460 cells. Cells were pretreated with **ZX-4C** (10, 20 μM) for 12 h and subsequently exposed to DSS (10 mg/mL) for an additional 6 h. Detection of cellular ROS levels using DCFH-DA fluorescent probe and observation of cellular ROS levels via live cell fluorescence microscopy, scale bars, 100 μm. (**B**–**D**) Detection of levels of GPx, SOD, and GSH/GSSG ratio in NCM460 cells. Cells were pretreatment with **ZX-4C** (10, 20 μM) for 24 h and afterward exposed to DSS (20 mg/mL) for an additional 12 h. (**E**–**G**) Analysis of the levels of pro-inflammatory cytokines IL-1β, IL-6, and TNF-α in NCM460 cells. Cells were pretreatment with **ZX-4C** (10, 20 μM) for 24 h and afterward exposed to DSS (20 mg/mL) for an additional 12 h. Values in (**B**–**G**) are shown as means ± SEM (n = 3). Statistical significance was determined by one-way ANOVA with Tukey’s multiple comparisons test: ^####^ *p* < 0.0001, ^###^ *p* < 0.001, vs. control group; **** *p* < 0.0001, *** *p* < 0.001, ** *p* < 0.01, * *p* < 0.05, vs. DSS group. (**B**), DSS group: *p* < 0.0001; 10 μM group: *p* = 0.0182; **20** μM group: *p* < 0.0001. (**C**), DSS group: *p* = 0.0002; 10 μM group: *p* = 0.034; 20 μM group: *p* = 0.0009. (**D**), DSS group: *p* < 0.0001; 10 μM group: *p* = 0.0024; 20 μM group: *p* = 0.0001. (**E**), DSS group: *p* < 0.0001; 10 μM group: *p* = 0.0014; 20 μM group: *p* < 0.0001. (**F**), DSS group: *p* < 0.0001; 10 μM group: *p* < 0.0001; 20 μM group: *p* < 0.0001. (**G**), DSS group: *p* < 0.0001; 10 μM group: *p* < 0.0001; 20 μM group: *p* < 0.0001.

Subsequently, the redox-sensitive fluorescent probe DCFH-DA was used to evaluate the antioxidant activity of **ZX-4C**. Treatment with DSS notably elevated ROS production, whereas pre-incubation with **ZX-4C** significantly reduced it (Figure 5A). Additionally, glutathione peroxidase (GPx) and superoxide dismutase (SOD), the activities of two representative enzymes were also measured. Pretreatment with **ZX-4C** remarkably restored the activities of GPx and SOD, thereby enhancing the antioxidant capacity of NCM460 cells (Figure 5B,C). The ratio of reduced glutathione (GSH) to oxidized glutathione (GSSG), an important indicator of the glutathione antioxidant system, was significantly reduced by treatment with DSS, and pretreatment of **ZX-4C** nearly normalized the ratio (Figure 5D). In the following step, enzyme-linked immunosorbent assay (ELISA) was utilized to quantitatively detect pro-inflammatory cytokines. Treatment with DSS elevated the levels of IL-1β, IL-6, and TNF-α, whereas pretreatment with **ZX-4C** significantly suppressed their secretion and nearly normalized the levels of these pro-inflammatory cytokines (Figure 5E–G). These results substantiated that **ZX-4C** protected NCM460 cells against DSS-induced injury by strengthening the antioxidant defense system and attenuating the inflammatory response.

### 3.5. ***ZX-4C*** Alleviated DSS-Induced Colitis in Mice

**Figure 6:**
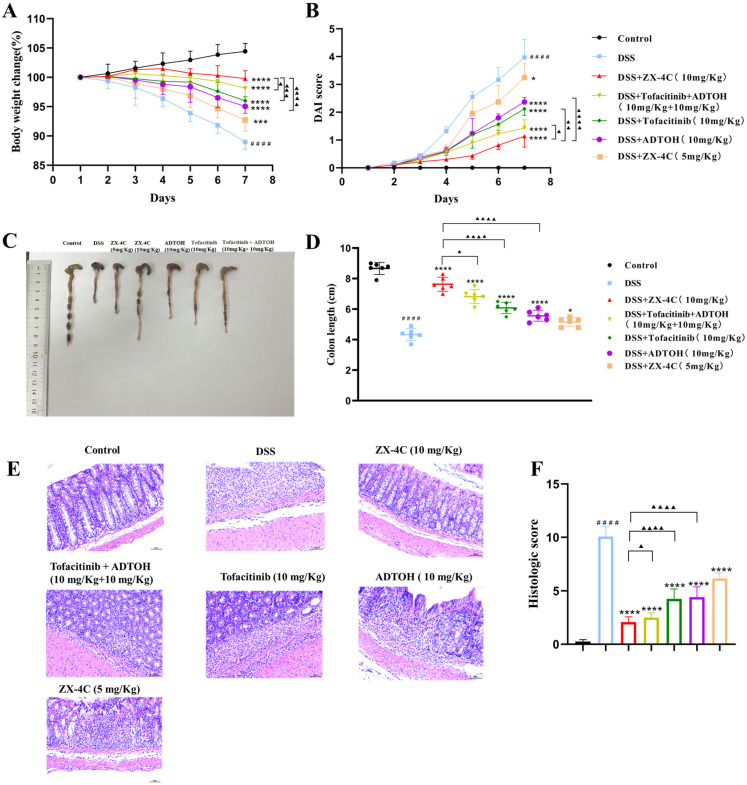
**ZX-4C** attenuates pathological symptoms of colitis in mice. The model was constructed by feeding with 3% (*w*/*v*) DSS drinking water for 7 consecutive days. Mice (n = 6) were injected intraperitoneally with therapeutic agent, **ZX-4C** (10 mg/kg or 5 mg/kg), tofacitinib (10 mg/kg), ADTOH (10 mg/kg) or tofacitinib plus ADTOH (10 mg/kg + 10 mg/kg) once daily for 10 days, starting before and continuing during DSS treatment. (**A**) Body weight change curves for each group. (**B**) DAI score curves for each group. (**C**) Representative imageries of colonic tissues from each group. (**D**) Measurement of colon length in each group. (**E**) Representative H&E staining images of colon sections (Scale bar = 50 μm). (**F**) Histologic scores of colon tissue. Values in (**A**,**B**,**D**,**F**) are presented as means ± SEM (n = 6). Statistical significance was determined by one-way ANOVA with Tukey’s multiple comparisons test: ^####^ *p* < 0.0001, vs. control group; **** *p* < 0.0001, *** *p* < 0.001, * *p* < 0.05, vs. DSS group; ^▲▲▲▲^ *p* < 0.0001, ^▲▲▲^ *p* < 0.001, ^▲▲^ *p* < 0.01, ^▲^ *p* < 0.05, vs. DSS+ **ZX-4C** group. (**A**), DSS model group: *p* < 0.0001; **ZX-4C** (10 mg/kg) group: *p* < 0.0001; tofacitinib + ADTOH (10 mg/kg + 10 mg/kg) group: *p* < 0.0001; tofacitinib (10 mg/kg) group: *p* < 0.0001; ADTOH (10 mg/kg) group: *p* < 0.0001; **ZX-4C** (5 mg/kg) group: *p* = 0.0006. (**B**), DSS model group: *p* < 0.0001; **ZX-4C** (10 mg/kg) group: *p* < 0.0001; tofacitinib + ADTOH (10 mg/kg + 10 mg/kg) group: *p* < 0.0001; tofacitinib (10 mg/kg) group: *p* < 0.0001; ADTOH (10 mg/kg) group: *p* < 0.0001; **ZX-4C** (5 mg/kg) group: *p* = 0.0352. (**D**), DSS model group: *p* < 0.0001; **ZX-4C** (10 mg/kg) group: *p* < 0.0001; tofacitinib + ADTOH (10 mg/kg + 10 mg/kg) group: *p* < 0.0001; tofacitinib (10 mg/kg) group: *p* < 0.0001; ADTOH (10 mg/kg) group: *p* < 0.0001; **ZX-4C** (5 mg/kg) group: *p* = 0.0151. (**F**), DSS model group: *p* < 0.0001; **ZX-4C** (10 mg/kg) group: *p* < 0.0001; tofacitinib + ADTOH (10 mg/kg + 10 mg/kg) group: *p* < 0.0001; tofacitinib (10 mg/kg) group: *p* < 0.0001; ADTOH (10 mg/kg) group: *p* < 0.0001; **ZX-4C** (5 mg/kg) group: *p* < 0.0001.

**Figure 7:**
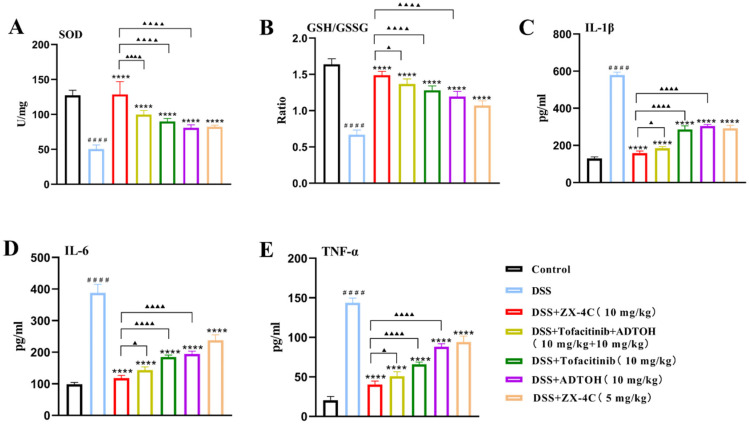
Measurement of antioxidant enzyme activities and pro-inflammatory cytokine levels in mice. Acute colitis was induced in mice with DSS and administered, **ZX-4C** (10 mg/kg or 5 mg/kg), tofacitinib (10 mg/kg), ADTOH (10 mg/kg) or tofacitinib plus ADTOH (10 mg/kg + 10 mg/kg) for 10 days. (**A**,**B**) Detection of levels of SOD and GSH/GSSG ratio in colon homogenates. (**C**–**E**) Analysis of the levels of pro-inflammatory cytokines IL-1β, IL-6, and TNF-α in colon homogenates. Values in (**A**–**E**) are presented as means ± SEM (n = 6). Statistical significance was determined by one-way ANOVA with Tukey’s multiple comparisons test: ^####^ *p* < 0.0001, vs. control group; **** *p* < 0.0001, vs. DSS group; ^▲▲▲▲^ *p* < 0.0001, ^▲^ *p* < 0.05, vs. DSS+ **ZX-4C** group. (**A**), DSS model group: *p* < 0.0001; **ZX-4C** (10 mg/kg) group: *p* < 0.0001; tofacitinib + ADTOH (10 mg/kg + 10 mg/kg) group: *p* < 0.0001; tofacitinib (10 mg/kg) group: *p* < 0.0001; ADTOH (10 mg/kg) group: *p* < 0.0001; **ZX-4C** (5 mg/kg) group: *p* < 0.0001. (**B**), DSS model group: *p* < 0.0001; **ZX-4C** (10 mg/kg) group: *p* < 0.0001; tofacitinib + ADTOH (10 mg/kg + 10 mg/kg) group: *p* < 0.0001; tofacitinib (10 mg/kg) group: *p* < 0.0001; ADTOH (10 mg/kg) group: *p* < 0.0001; **ZX-4C** (5 mg/kg) group: *p* < 0.0001. (**C**), DSS model group: *p* < 0.0001; **ZX-4C** (10 mg/kg) group: *p* < 0.0001; tofacitinib + ADTOH (10 mg/kg + 10 mg/kg) group: *p* < 0.0001; tofacitinib (10 mg/kg) group: *p* < 0.0001; ADTOH (10 mg/kg) group: *p* < 0.0001; **ZX-4C** (5 mg/kg) group: *p* < 0.0001. (**D**), DSS model group: *p* < 0.0001; **ZX-4C** (10 mg/kg) group: *p* < 0.0001; tofacitinib + ADTOH (10 mg/kg + 10 mg/kg) group: *p* < 0.0001; tofacitinib (10 mg/kg) group: *p* < 0.0001; ADTOH (10 mg/kg) group: *p* < 0.0001; **ZX-4C** (5 mg/kg) group: *p* < 0.0001. (**E**), DSS model group: *p* < 0.0001; **ZX-4C** (10 mg/kg) group: *p* < 0.0001; tofacitinib + ADTOH (10 mg/kg + 10 mg/kg) group: *p* < 0.0001; tofacitinib (10 mg/kg) group: *p* < 0.0001; ADTOH (10 mg/kg) group: *p* < 0.0001; **ZX-4C** (5 mg/kg) group: *p* < 0.0001.

Inspired by the in vitro experimental discoveries, we next investigated the in vivo efficacy of **ZX-4C** with a mouse model of acute UC. The model was established by feeding with 3% (*w*/*v*) DSS drinking water for 7 consecutive days. The severity of colitis was evaluated by measuring body weight, disease activity index (DAI), and colon length. As shown in Figure 6A–D, mice in the DSS group exhibited severe symptoms of colitis, characterized by reduced body weight, high DAI scores, and shortened colon length. The experimental results indicated that the pretreatment of **ZX-4C** significantly mitigated these symptoms, including increased body weight, attenuated DAI scores, and recovered colon length. Pretreatment with **ZX-4C** had a significant therapeutic effect, more effective than ADTOH, tofacitinib, and the combination of tofacitinib and ADTOH at the same dosage (10 mg/kg). Histopathological examination of hematoxylin and eosin (H&E)—stained colon tissue sections further revealed that **ZX-4C** mitigated the tissue damage and inflammatory cell infiltration induced by DSS (Figure 6E,F). Moreover, **ZX-4C** enhanced SOD activity and GSH/GSSG ratio in colon homogenates, demonstrating that **ZX-4C** restored the DSS-induced decrease in antioxidant activity (Figure 7A,B). Subsequently, we further evaluated the levels of pro-inflammatory factors (IL-1β, IL-6, and TNF-α) in colon homogenates using ELISA. The results revealed that the DSS model group showed significantly elevated levels of pro-inflammatory factors in colon homogenates, whereas administration of **ZX-4C** significantly diminished these inflammatory markers, demonstrating a potent anti-inflammatory effect (Figure 7C–E). Altogether, these results indicated that **ZX-4C** significantly attenuated DSS-induced acute colitis symptoms in mice, demonstrating its potent in vivo efficacy. Further analysis revealed that tofacitinib exerted a stronger colitis therapeutic effect compared to ADTOH at the same dosage.

## 4. Conclusions

In summary, a series of novel tofacitinib–ADTOH hybrids were designed and synthesized as potential therapeutic agents for ulcerative colitis. All hybrids demonstrated enhanced cytoprotective effects against DSS-induced damage in NCM460 cells. Notably**, ZX-4C**, which demonstrated the most prominent effect, protected NCM460 cells against DSS-induced injury by enhancing the antioxidant defense system and attenuating the inflammatory response. Further in vivo studies revealed that the pretreatment with **ZX-4C** significantly mitigated the symptoms of DSS-induced colitis in mice more effectively than treatment with tofacitinib or ADTOH alone. Although **ZX-4C** demonstrated better cytoprotective effects as well as therapeutic effects in colitis, its long-term safety, and possible off-target effects need to be further validated. Altogether, the molecular hybridization of tofacitinib and H_2_S donors represents a promising strategy for the treatment of ulcerative colitis and provides novel perspectives on the development of drugs for other inflammatory diseases.

## Data Availability

The data presented in this study are available in the article and Supplementary Materials.

## Supplementary Materials

The following supporting information can be downloaded at: https://www.mdpi.com/article/10.3390/antiox14030325/s1, Supplementary information includes drug release assays for **ZX-4C** and structural characterization data for all compounds; Figure S1. Validation of esterase-responsive drug release from compound **ZX-4C**.

## Abbreviations

ADTOH: 5-(4-hydroxyphenyl)-3H-1, 2-dithio-3-thioone; CCK-8, Cell Counting Kit-8; DAI, disease activity index; DSS, dextran sulfate sodium; ELISA, enzyme-linked immunosorbent assay; GSH, reduced glutathione; GSH-Px, glutathione peroxidase; GSSG, oxidized glutathione; H&E, hematoxylin and eosin; ROS, reactive oxygen species; SOD, superoxide dismutase; UC, ulcerative colitis.
