# Supplementary material for: Discovery of Novel Tofacitinib–ADTOH Molecular Hybridization Derivatives for the Treatment of Ulcerative Colitis
Source: Antioxidants (Basel). 2025 Mar 8;14(3):325. doi: 10.3390/antiox14030325 (PMC11939234; doi:10.3390/antiox14030325)

Supplementary Information

Discovery of novel Tofacitinib/ADTOH molecular hybridization derivatives for the treatment of ulcerative colitis

Yi Mou [1] †, Shuai Wen [1, 2] †, Yan Wang [1], Yao Zhao [1], Ying-Ping Li [1], Hong-Kai Sha [1,] Li-Juan Gui [1], Zheng-Yu Jiang [2, *], Xiang-Ming Xu [1, *]

[1] College of Pharmacy and Chemistry & Chemical Engineering, Taizhou University, Taizhou 225300, China; mouyicpu@163.com (Y.M.); wen15298503462@126.com (S.W.); wangyan20221122@163.com (Y.W.); 15996256893@163.com (Y.Z.); 2024110049@tzu.edu.cn(Y.-P.L.); shahongkaitzxy@163.com (H.-K.S.); guilijuan12@163.com (L.-J.G.)

[2] Jiangsu Key Laboratory of Drug Design and Optimization, China Pharmaceutical University, Nanjing 210009, China;

[*] Correspondence: yzxmxu@163.com (X.-M.X.); jiangzhengyucpu@163.com (Z.-Y.J.)

[†] These authors contributed equally to this work.

Figure S1. Validation of esterase-responsive drug release from compound **ZX-4C**.

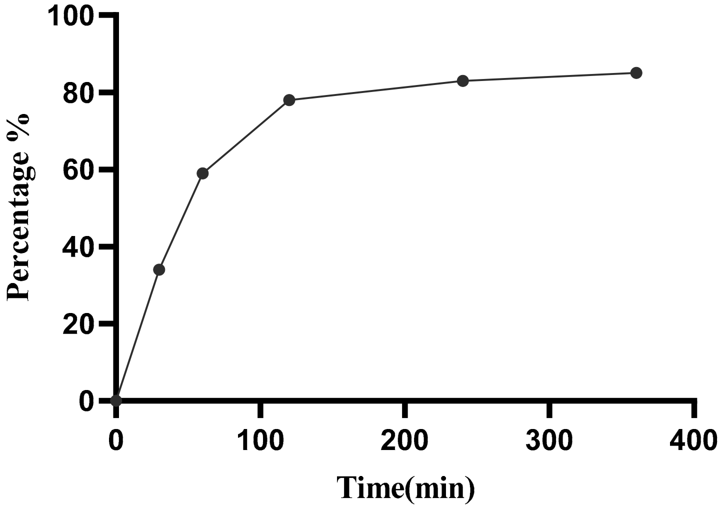

Structure characterization of intermediates.

1. ^{1}H NMR (300 MHz, DMSO-d_6) for **3a**

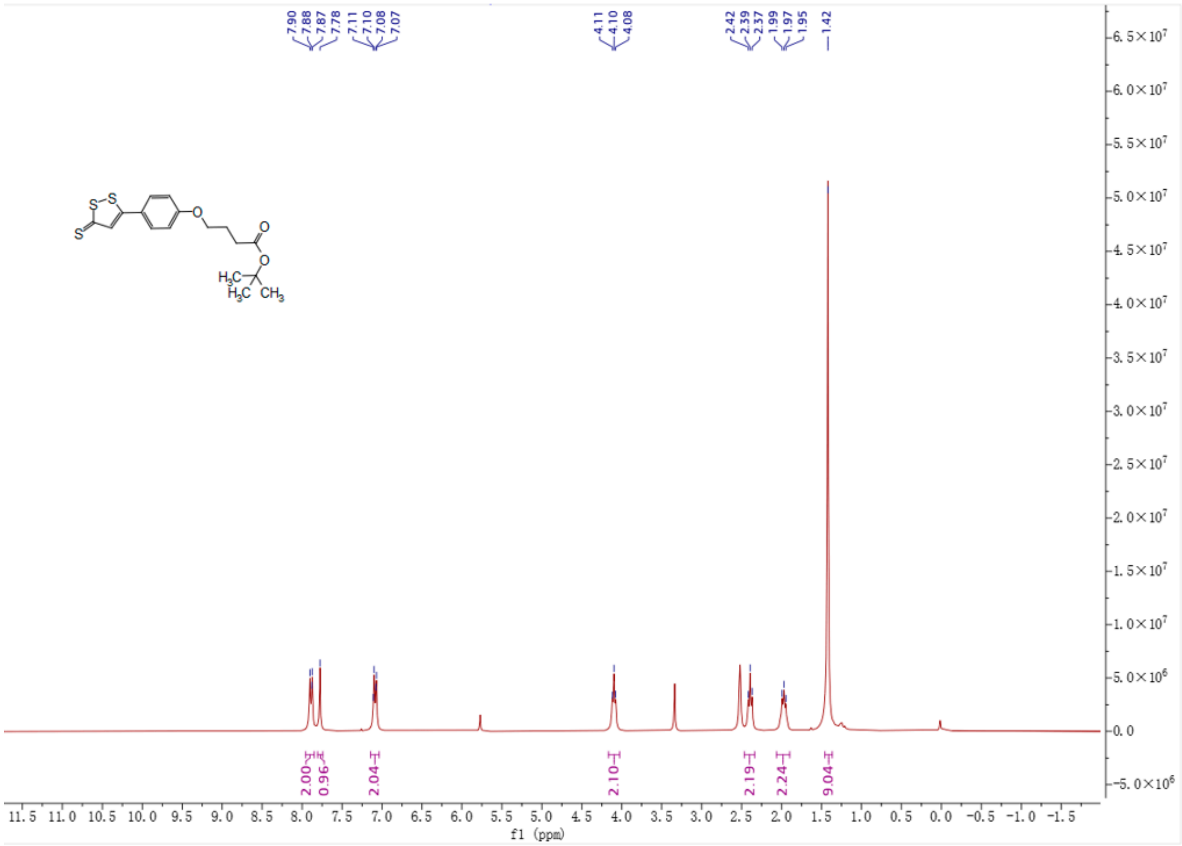

2. ^{1}H NMR (300 MHz, Chloroform-d) for **3b**

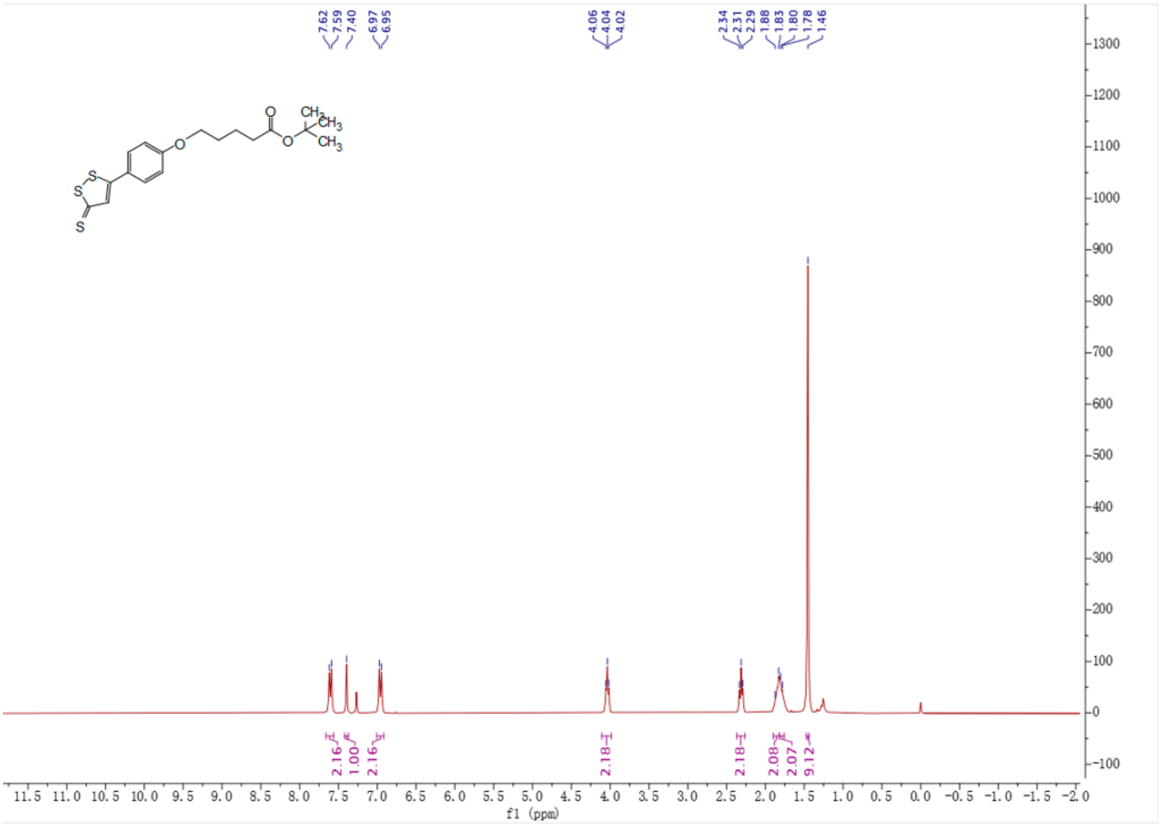

3. ^{1}H NMR (300 MHz, Chloroform-*d*) for **3c**

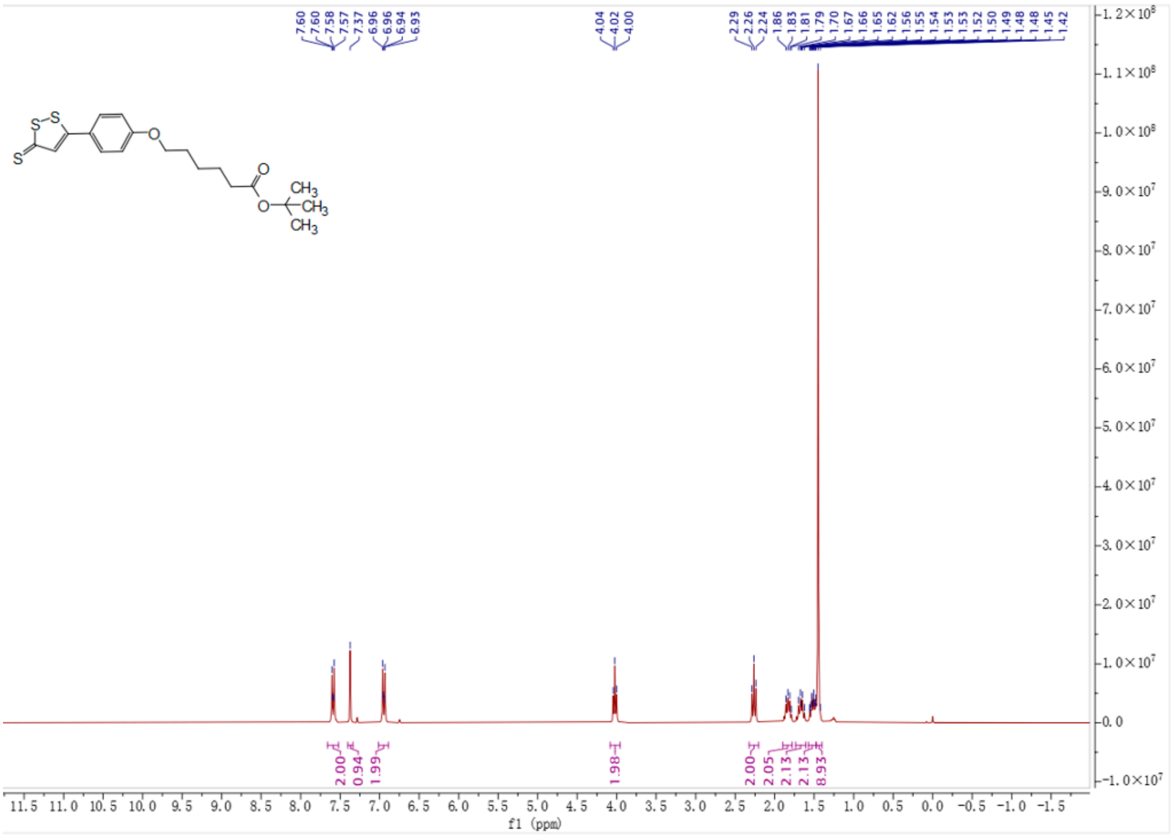

4. ^{1}H NMR (300 MHz, DMSO-d_6) for **4a**

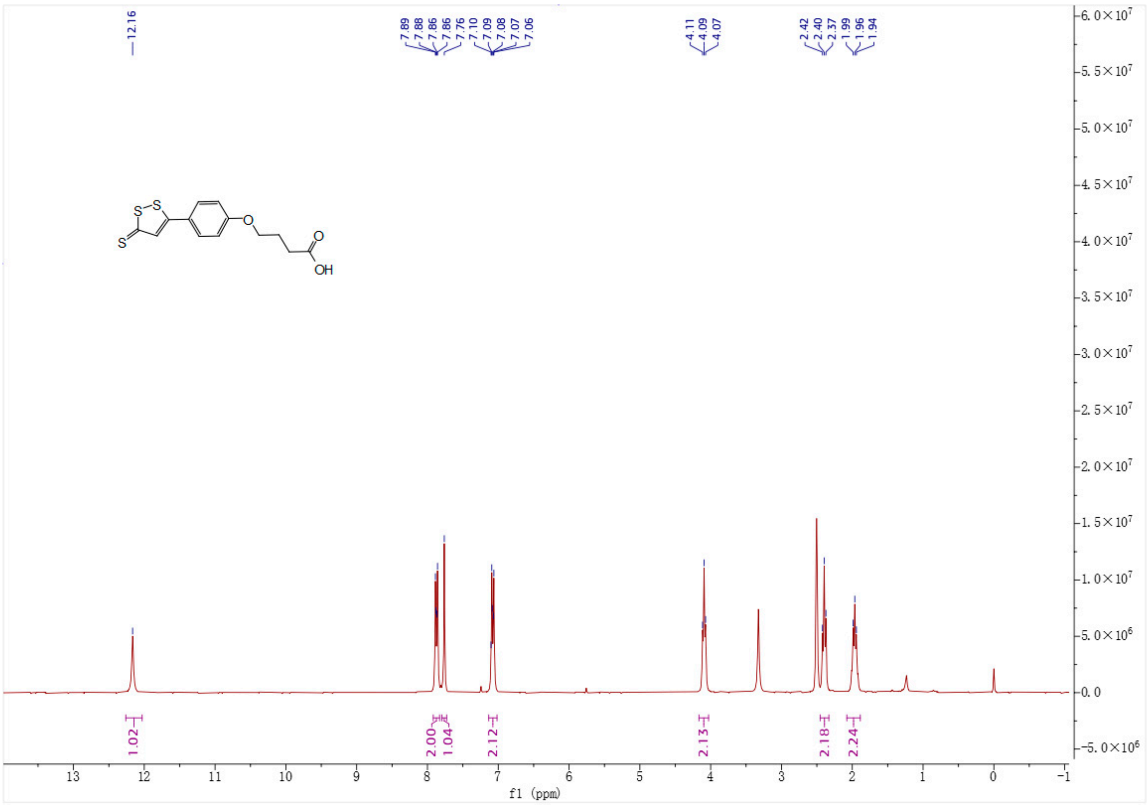

5. ^{1}H NMR (300 MHz, DMSO-d_6) for **4b**

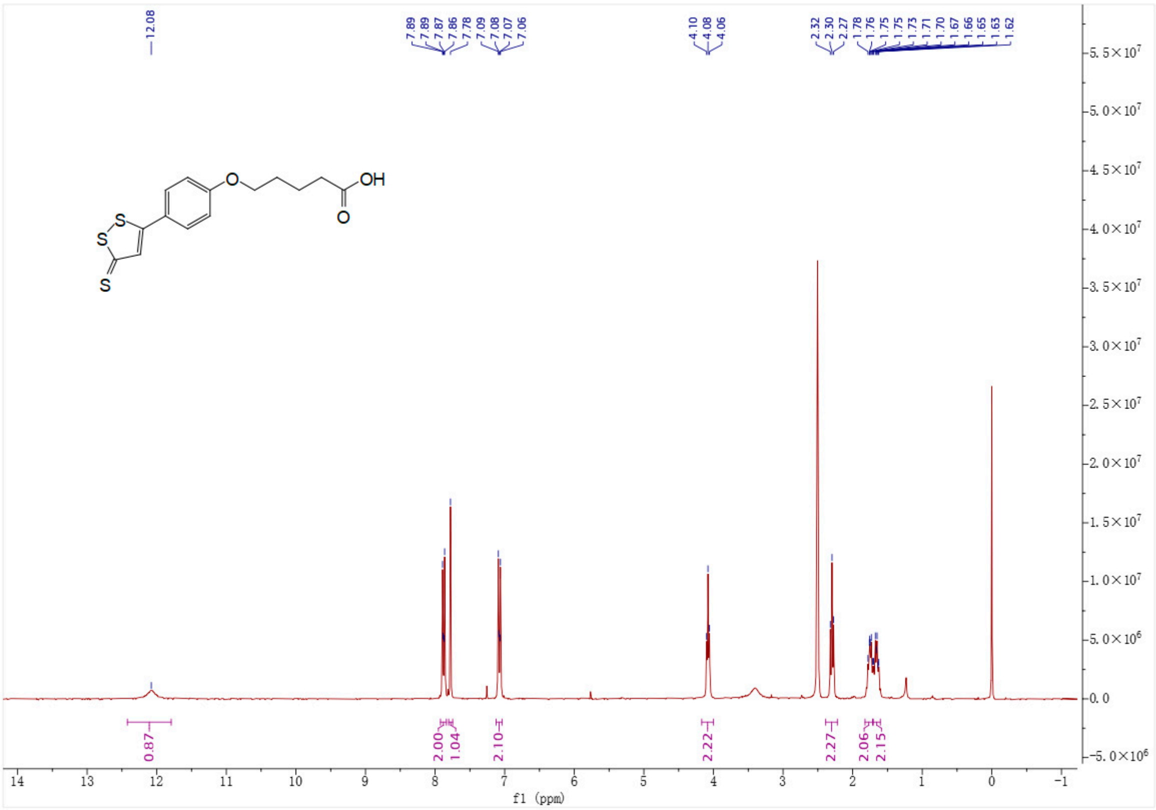

6. ^{1}H NMR (300 MHz, DMSO-d_6) for **4c**

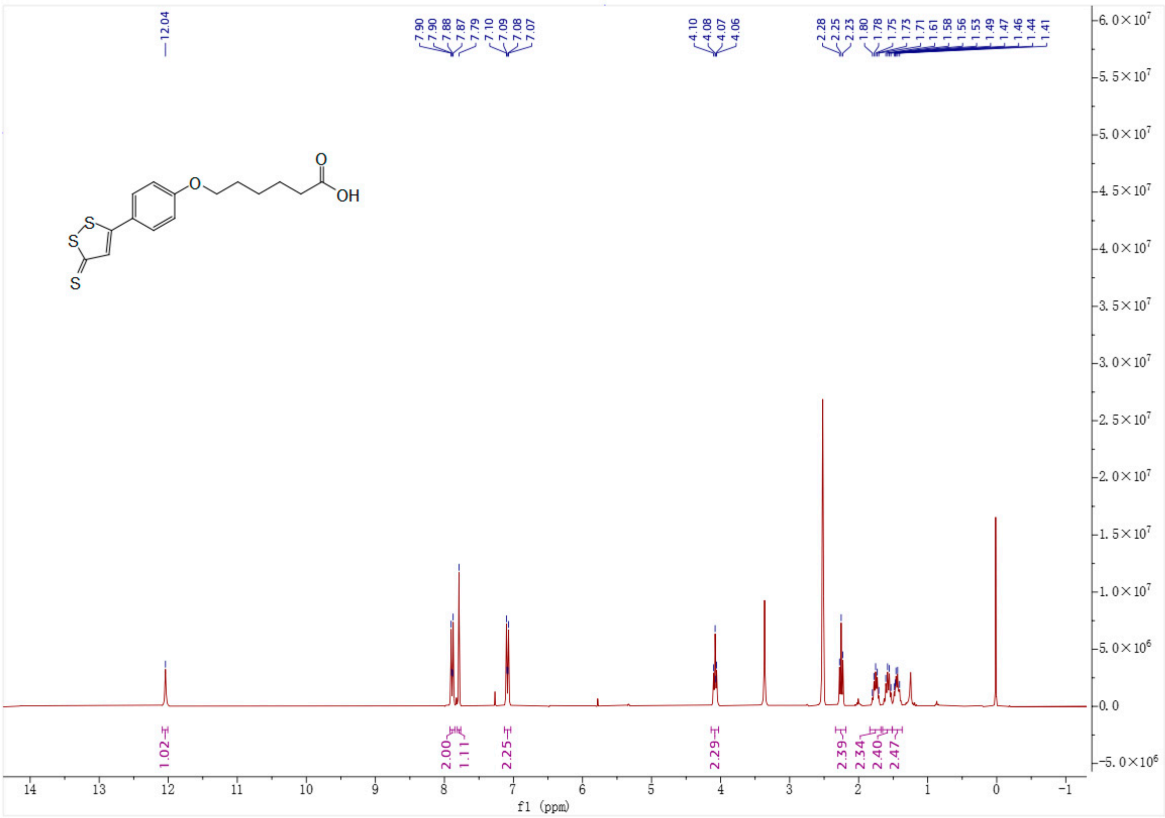

7. ^{1}H NMR (300 MHz, Chloroform-d) for **7**

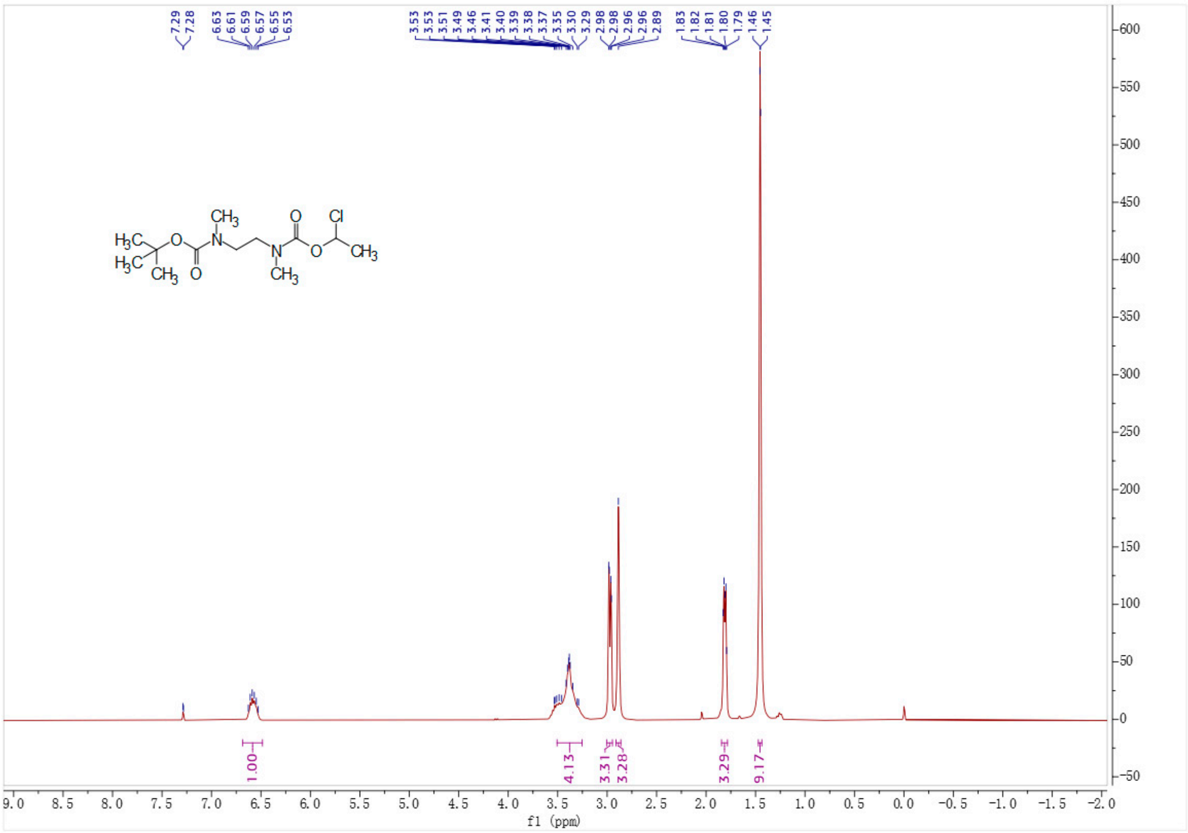

8. ^{1}H NMR (300 MHz, Chloroform-*d*) for **8a**

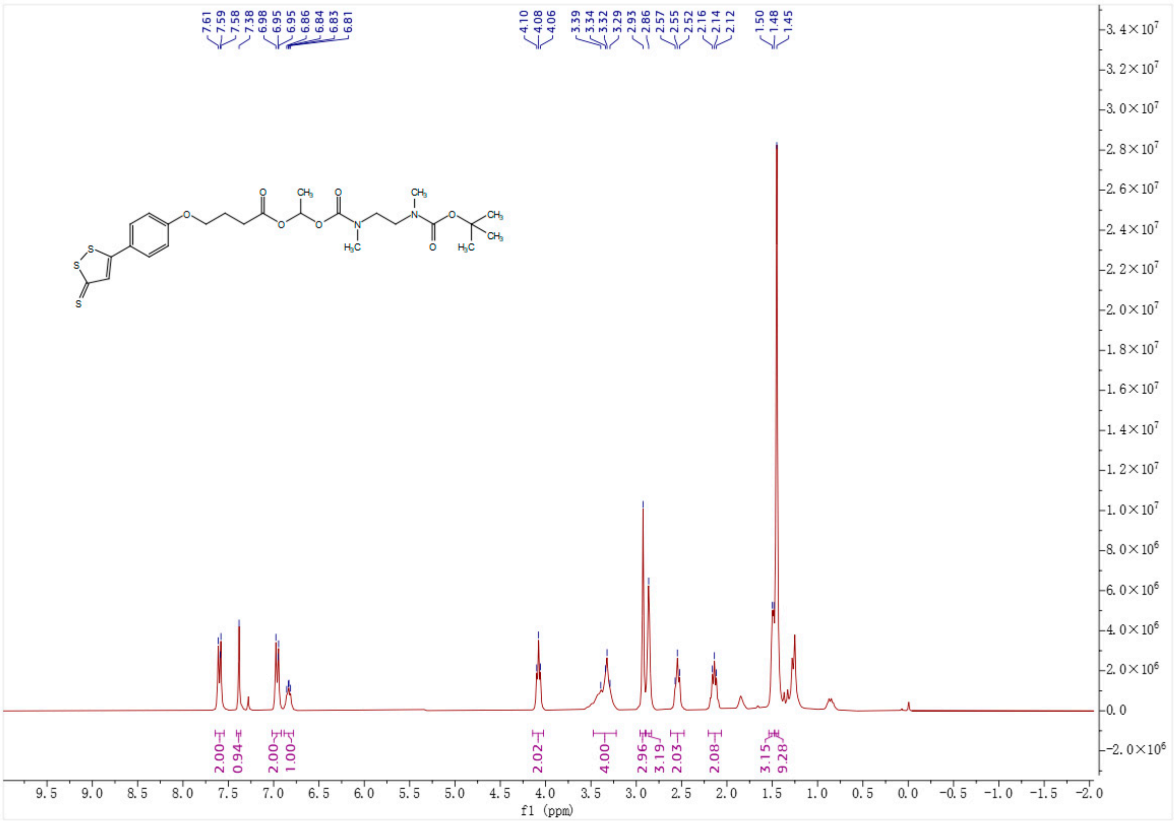

9. ^{1}H NMR (300 MHz, Chloroform-*d*) for **8b**

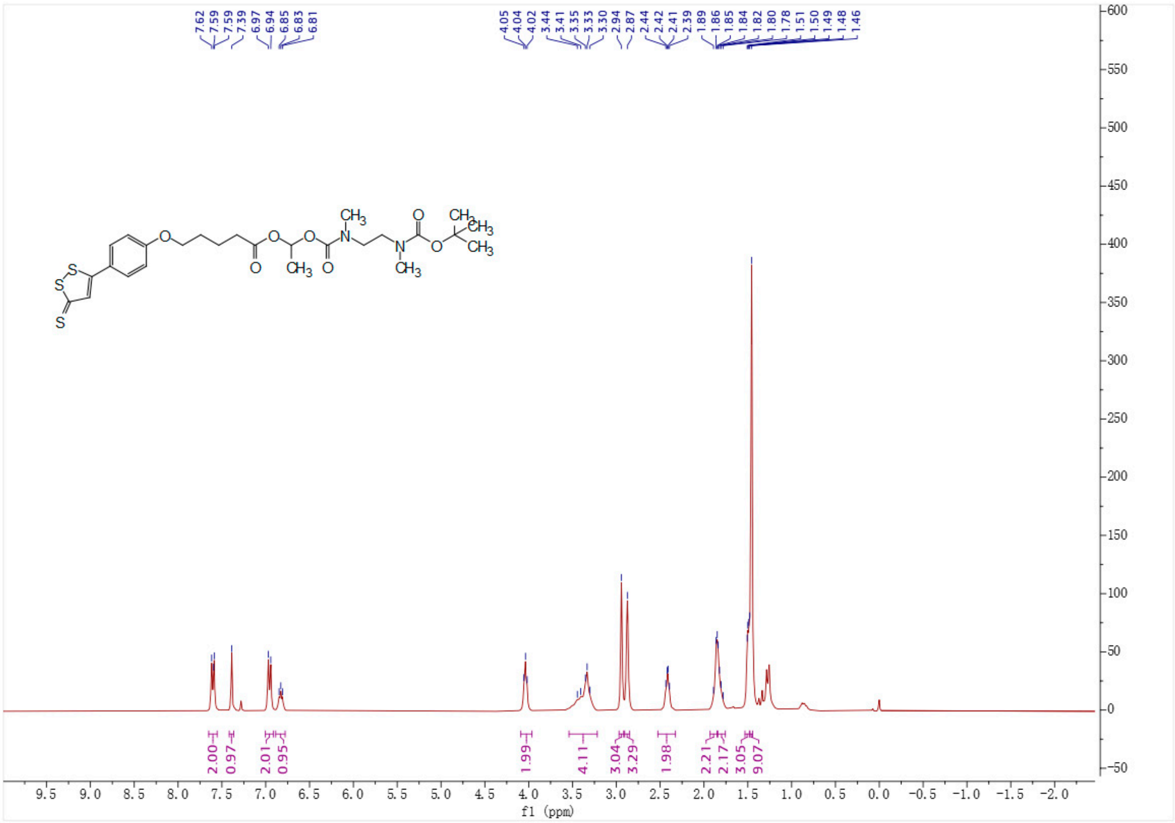

10. ^{1}H NMR (300 MHz, Chloroform-*d*) for **8c**

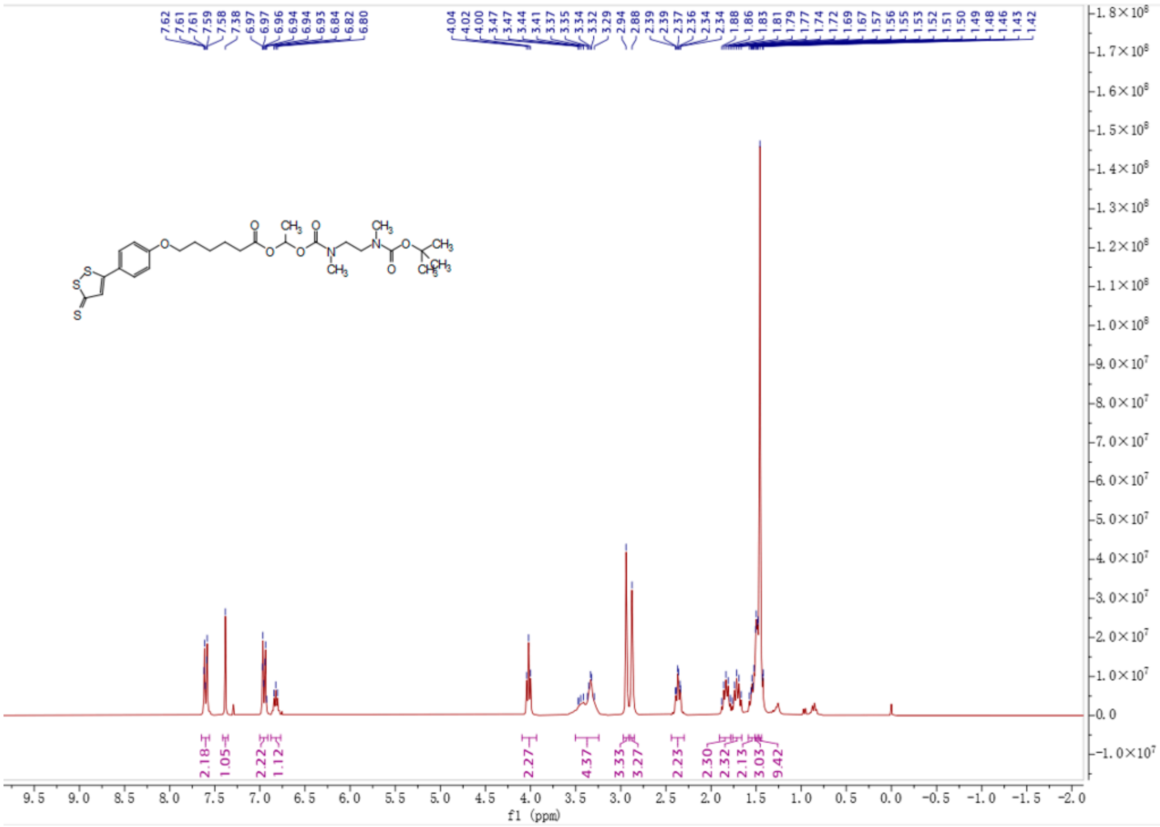

Structure characterization of target compounds.

11. ^{1}H NMR (300 MHz, Chloroform-*d*), ^{13}C NMR (151 MHz, $CDCl_3$), HRMS, and HPLC for **ZX-4C**

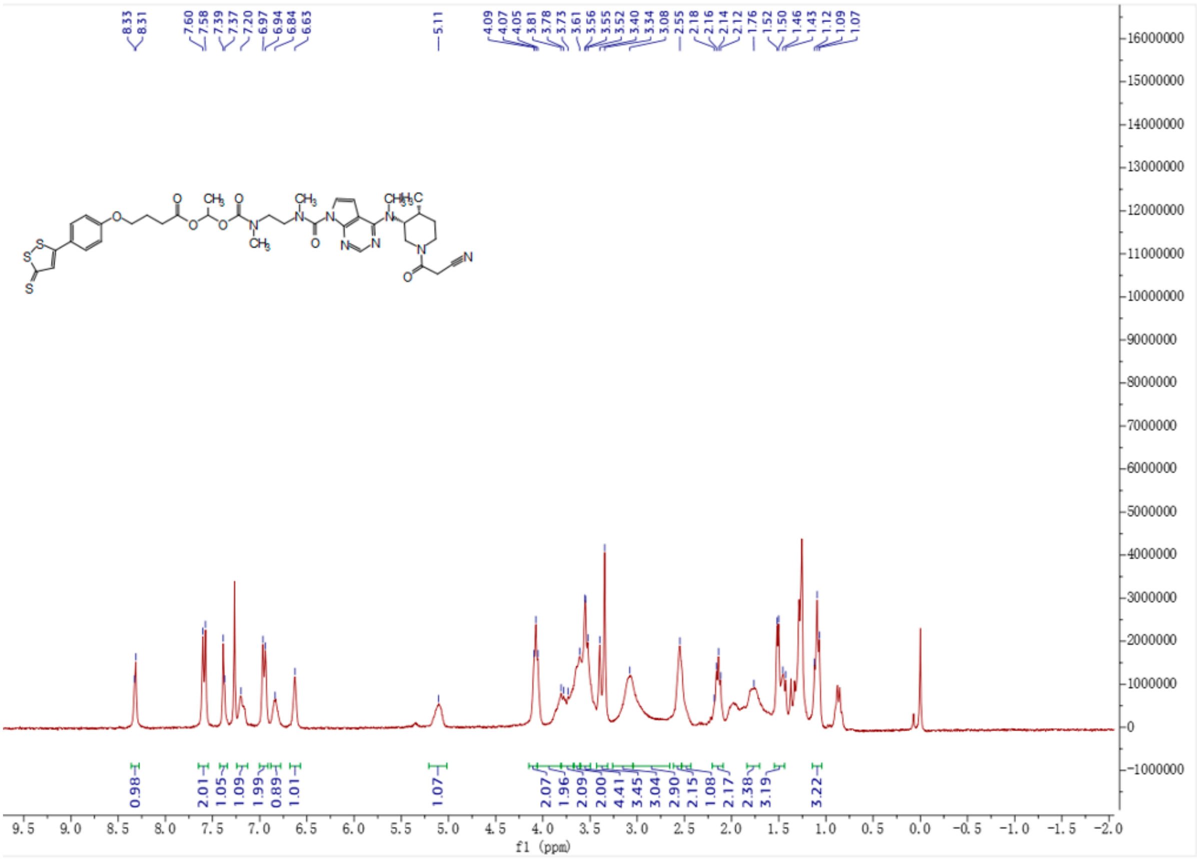

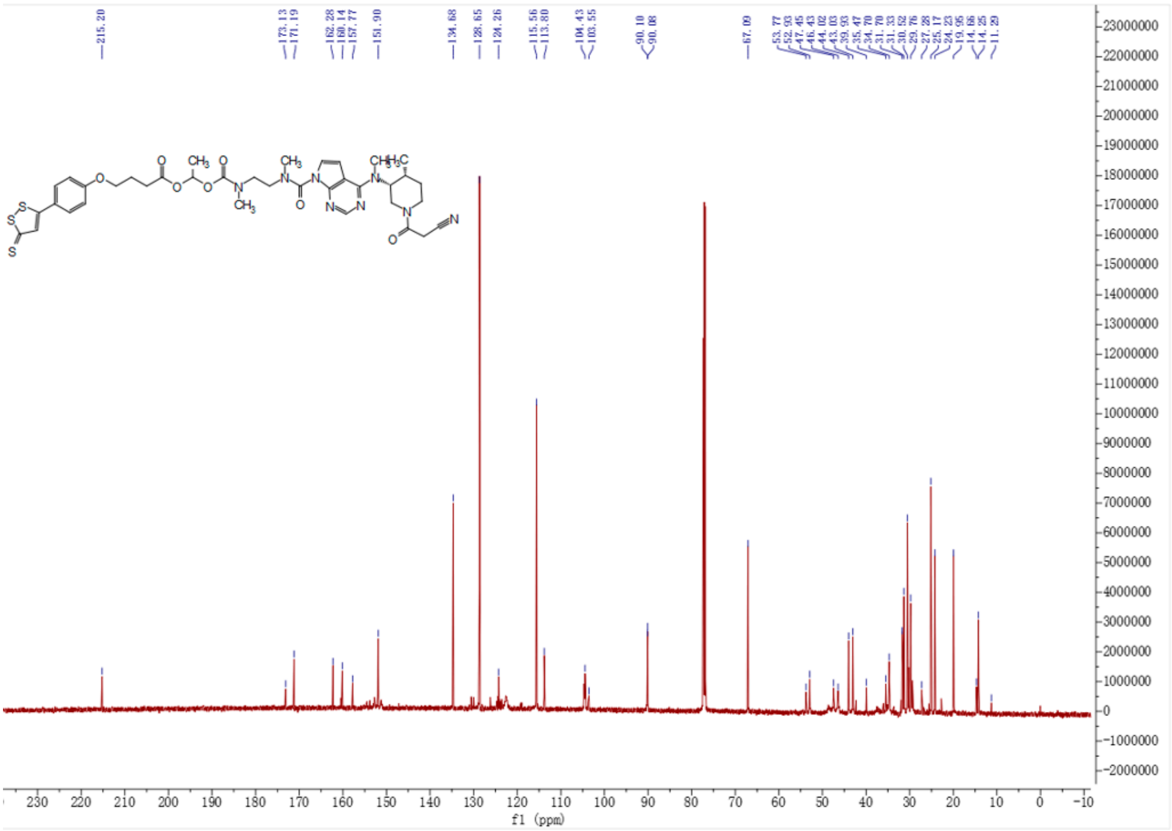

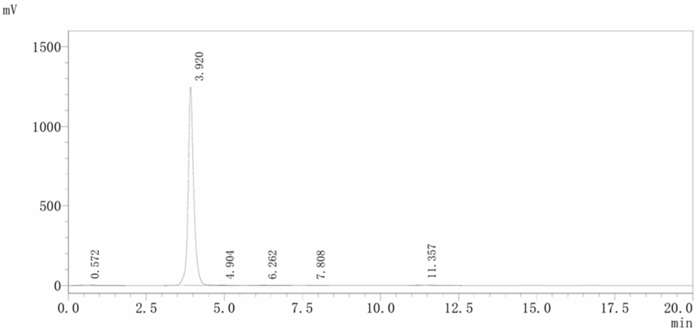

<Peak Table>
Detector A 254 nM

Number	Retention time	Area	Height	Area%
1	0.572	126659	4400	0.802
2	3.920	15499490	1247587	98.126
3	4.904	9243	723	0.059
4	6.262	29876	1140	0.189
5	7.808	5081	239	0.032
6	11.357	125187	4328	0.793
Total		15795536	1258417	100.000

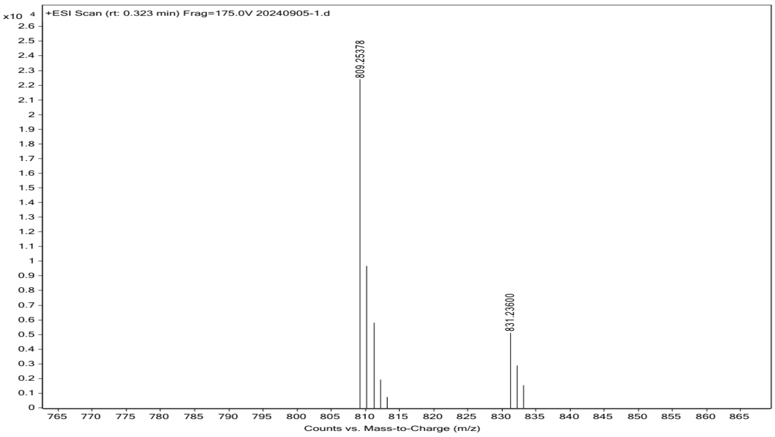

12. ^{1}H NMR (300 MHz, Chloroform-*d*), ^{13}C NMR (75 MHz, CDCl$_3$), HRMS, and HPLC for **ZX-5C**

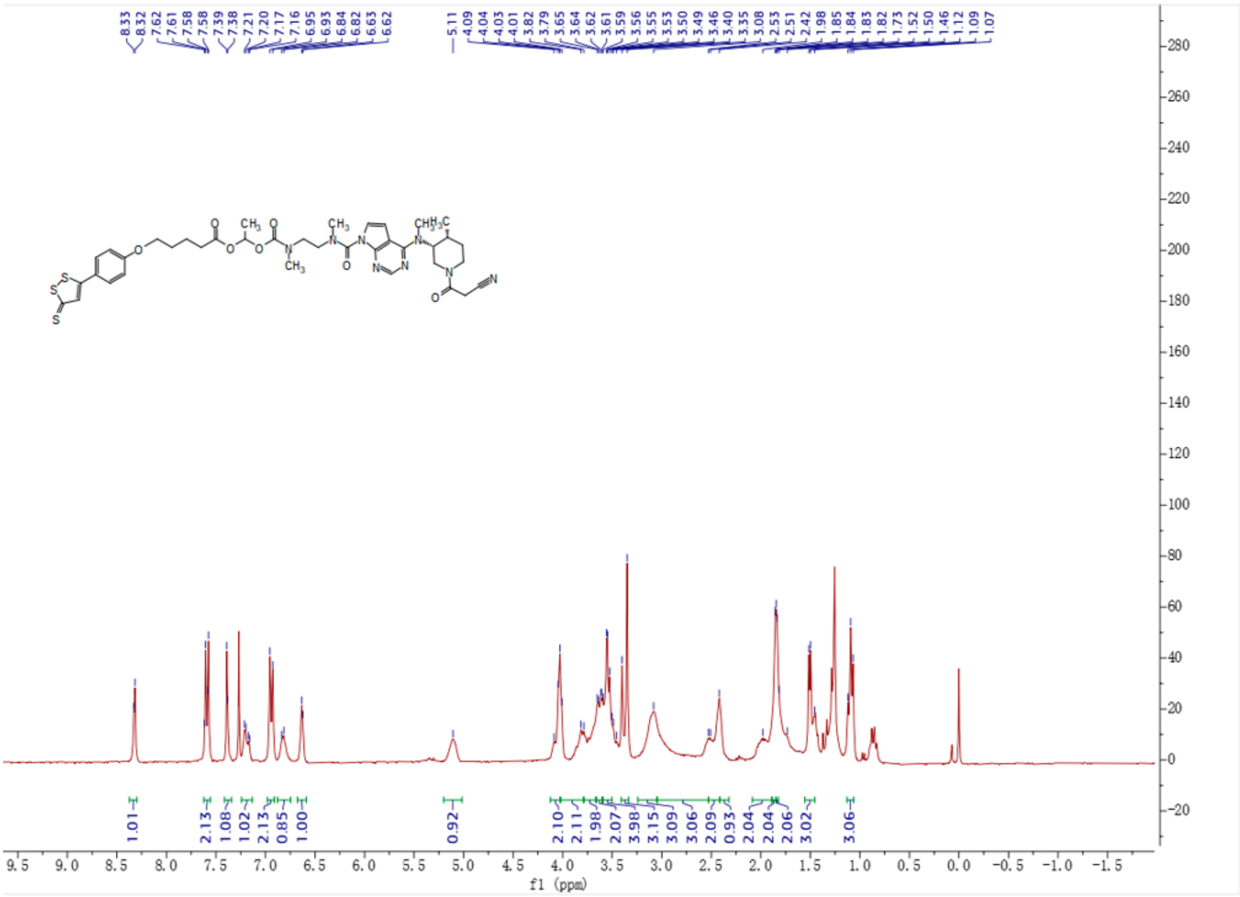
8.33
8.32
7.62
7.61
7.58
7.58
7.39
7.38
7.21
7.20
7.17
7.16
6.95
6.93
6.84
6.82
6.63
6.62
5.11
4.09
4.04
4.03
4.01
3.82
3.79
3.65
3.64
3.62
3.61
3.59
3.56
3.55
3.53
3.50
3.49
3.46
3.40
3.35
3.08
2.53
2.51
2.42
1.98
1.85
1.84
1.83
1.82
1.73
1.52
1.50
1.46
1.12
1.09
1.07
1.01
2.13
1.08
1.02
2.13
0.85
1.00
0.92
2.10
2.11
1.98
2.07
3.98
3.15
3.09
3.06
2.09
0.93
2.04
2.04
2.06
3.02
3.06
f1 (ppm)

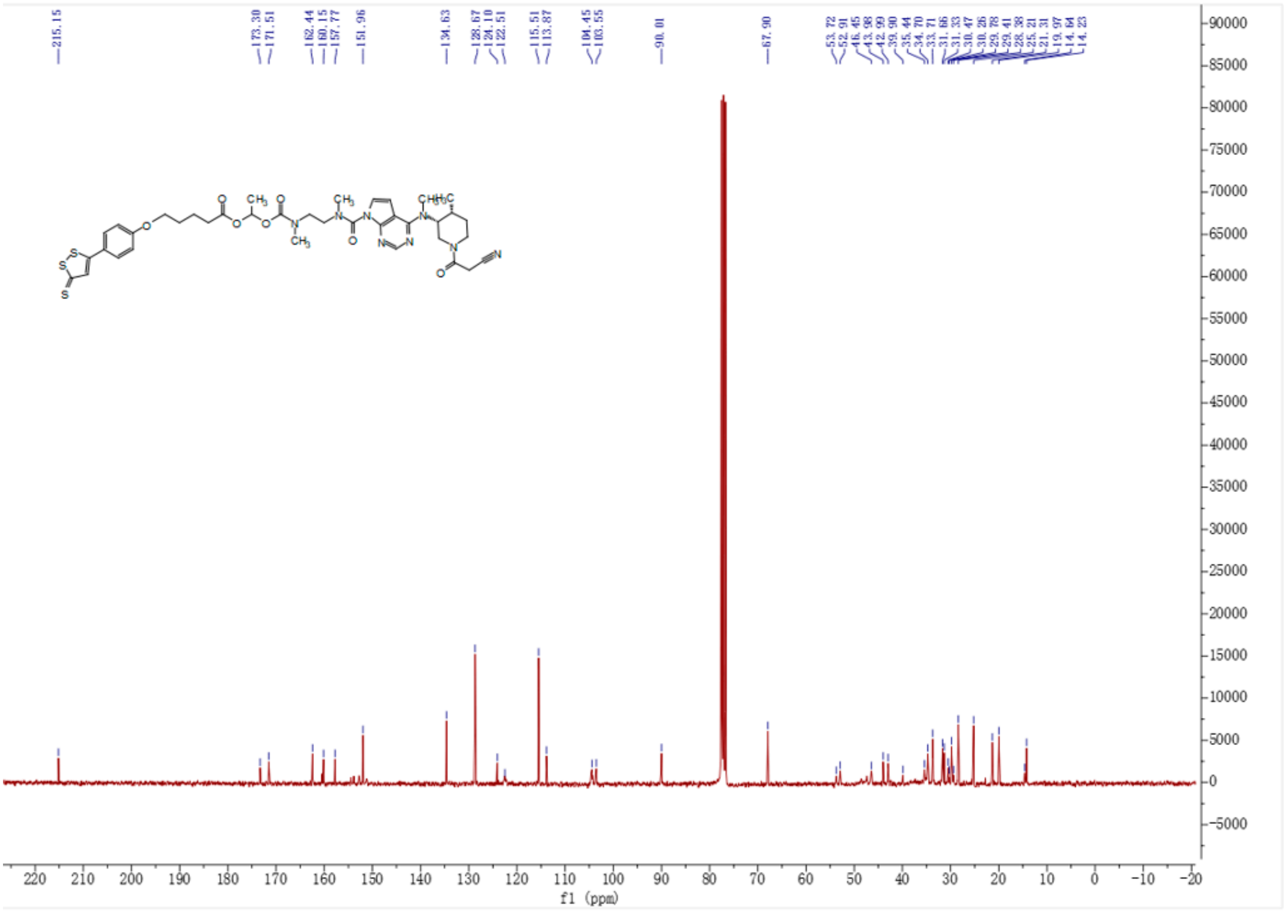

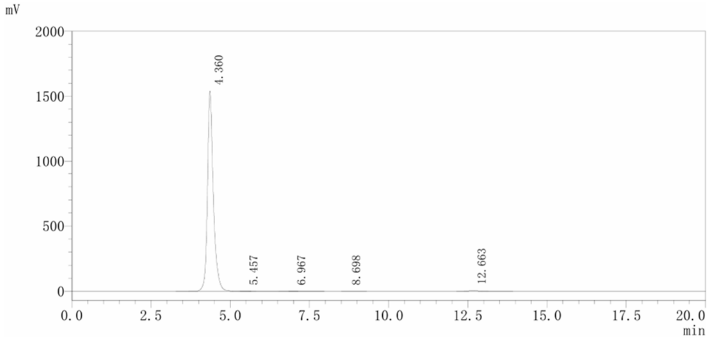

<Peak Table>
Detector A 254 nM

Number	Retention time	Area	Height	Area%
1	4.360	19980326	1540307	98.946
2	5.457	12452	944	0.062
3	6.967	39155	1377	0.194
4	8.698	5476	249	0.027
5	12.663	155787	5102	0.771
Total		20193195	1547979	100.000

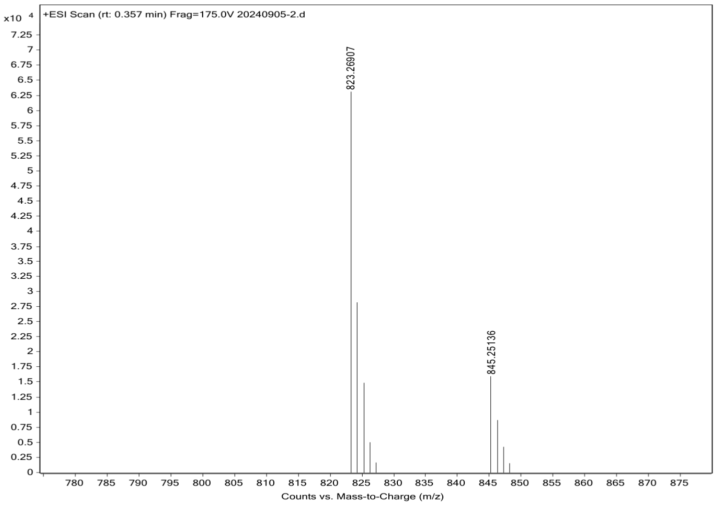

13. ^{1}H NMR (300 MHz, Chloroform-*d*), ^{13}C NMR (75 MHz, CDCl$_3$), HRMS, and HPLC for **ZX-6C**

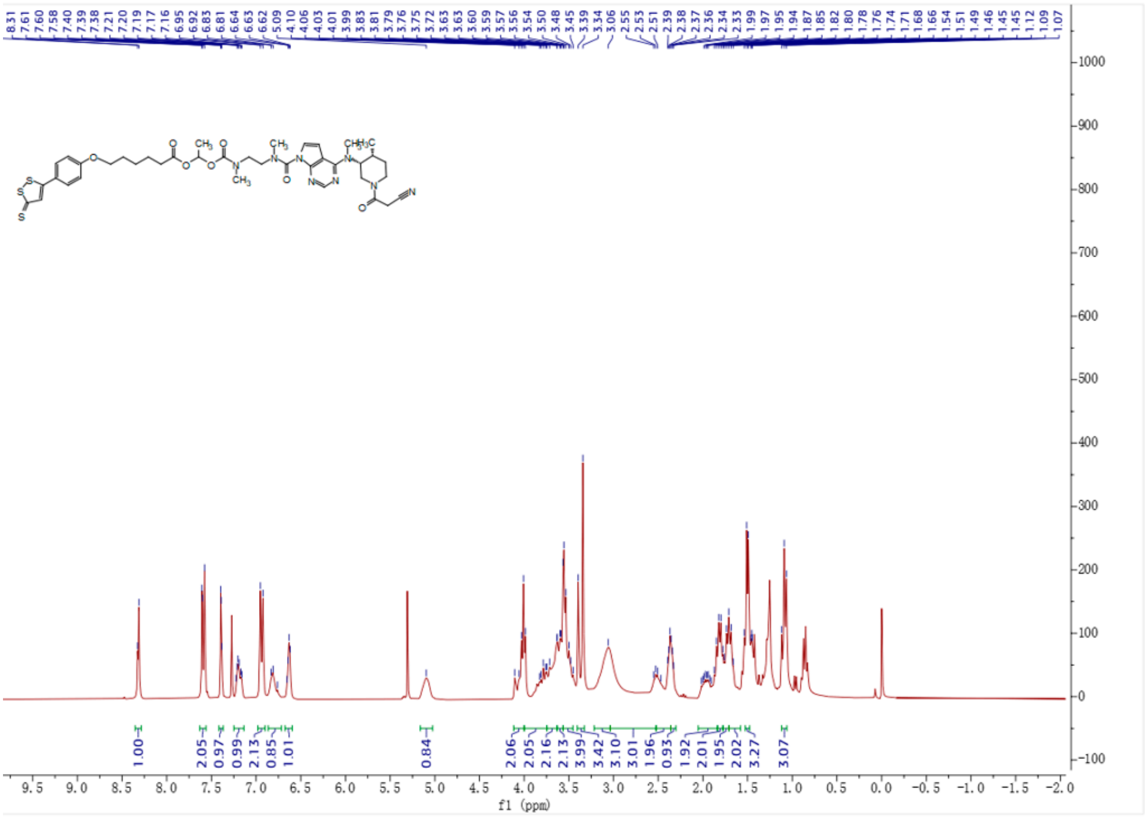

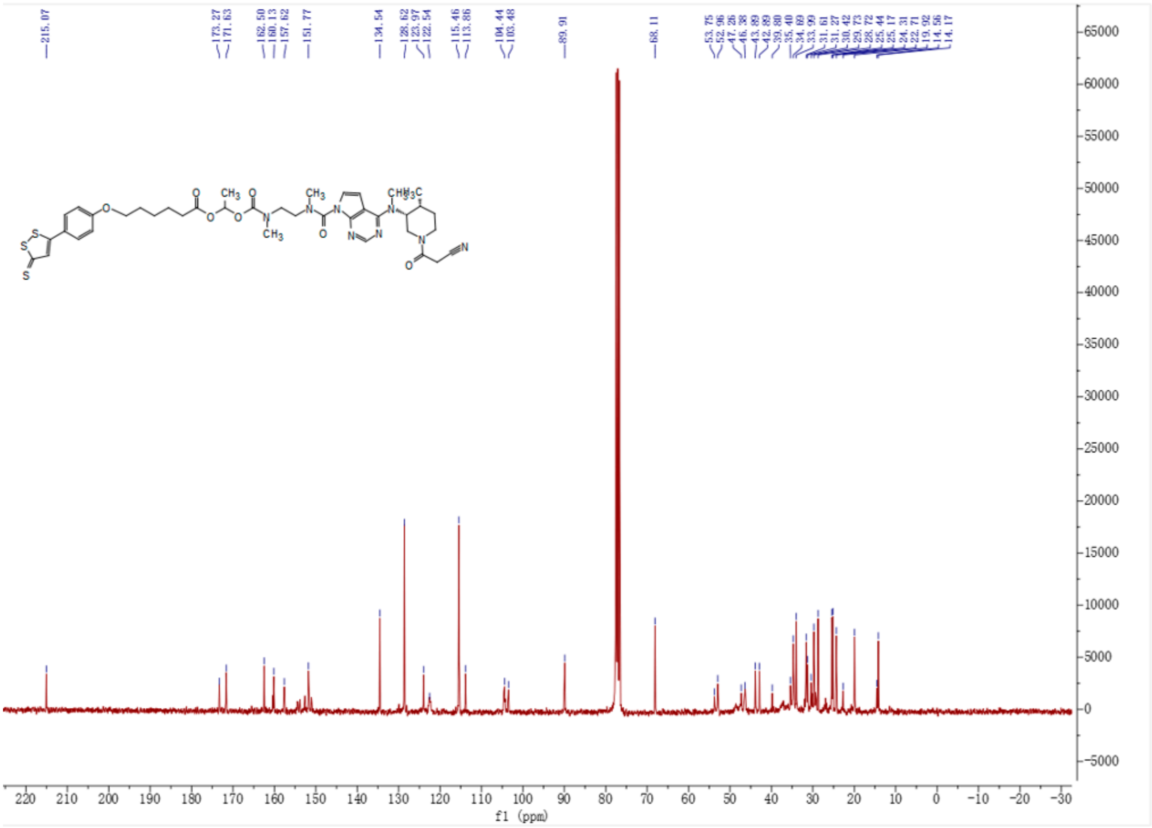

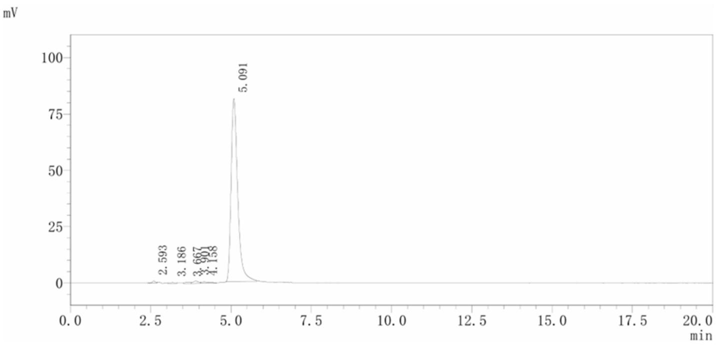

<Peak Table>
Detector A 254 nM

Number	Retention time	Area	Height	Area%
1	2.593	7519	953	0.624
2	3.186	4946	357	0.410
3	3.667	5515	404	0.458
4	3.901	11776	1008	0.977
5	4.158	12767	647	1.059
6	5.091	1162503	81194	96.471
Total		1205026	84563	100.000

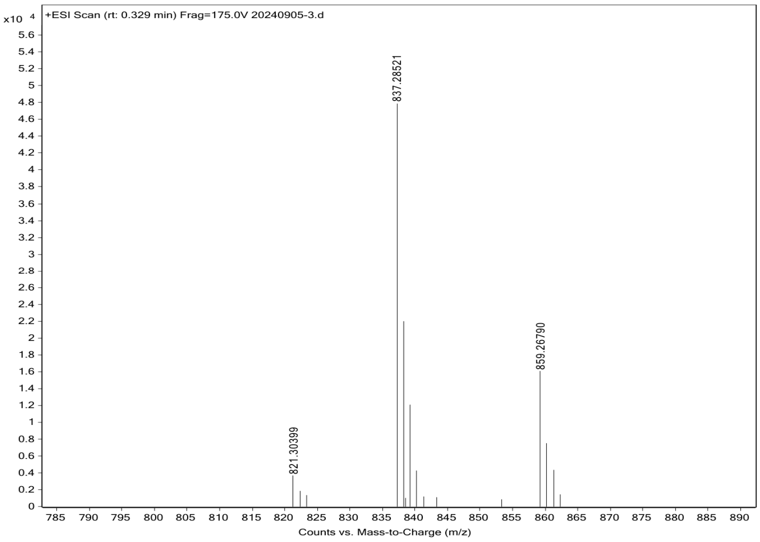

Supplement: Supplementary file 1 [file antioxidants-14-00325-s001.zip › antioxidants-3489026-supplementary.pdf]
